# Cost-effectiveness analysis of reflex testing for Lynch syndrome in women with endometrial cancer in the UK setting

**DOI:** 10.1371/journal.pone.0221419

**Published:** 2019-08-30

**Authors:** Tristan M. Snowsill, Neil A. J. Ryan, Emma J. Crosbie, Ian M. Frayling, D. Gareth Evans, Chris J. Hyde

**Affiliations:** 1 Health Economics Group, University of Exeter Medical School, University of Exeter, Exeter, Devon, United Kingdom; 2 Division of Cancer Sciences, Faculty of Biology and Health, University of Manchester, Manchester, United Kingdom; 3 Division of Evolution and Genomic Medicine, Faculty of Biology and Health, University of Manchester, Manchester, United Kingdom; 4 Directorate of Gynaecology, St Mary’s Hospital, Manchester University NHS Foundation Trust, Manchester, United Kingdom; 5 Inherited Tumour Syndromes Research Group, Institute of Cancer and Genetics, Cardiff University, Cardiff, United Kingdom; 6 Manchester Centre for Genomic Medicine, Manchester University NHS Foundation Trust, Manchester, United Kingdom; 7 Exeter Test Group, University of Exeter Medical School, University of Exeter, Exeter, Devon, United Kingdom; Leiden University Medical Centre, NETHERLANDS

## Abstract

**Background:**

Lynch syndrome is a hereditary cancer syndrome caused by constitutional pathogenic variants in the DNA mismatch repair (MMR) system, leading to increased risk of colorectal, endometrial and other cancers. The study aimed to identify the incremental costs and consequences of strategies to identify Lynch syndrome in women with endometrial cancer.

**Methods:**

A decision-analytic model was developed to evaluate the relative cost-effectiveness of reflex testing strategies for identifying Lynch syndrome in women with endometrial cancer taking the NHS perspective and a lifetime horizon. Model input parameters were sourced from various published sources. Consequences were measured using quality-adjusted life years (QALYs). A cost-effectiveness threshold of £20 000/QALY was used.

**Results:**

Reflex testing for Lynch syndrome using MMR immunohistochemistry and *MLH1* methylation testing was cost-effective versus no testing, costing £14 200 per QALY gained. There was uncertainty due to parameter imprecision, with an estimated 42% chance this strategy is not cost-effective compared with no testing. Age had a significant impact on cost-effectiveness, with testing not predicted to be cost-effective in patients aged 65 years and over.

**Conclusions:**

Testing for Lynch syndrome in younger women with endometrial cancer using MMR immunohistochemistry and *MLH1* methylation testing may be cost-effective. Age cut-offs may be controversial and adversely affect implementation.

## Introduction

Lynch syndrome (LS) is a hereditary cancer syndrome caused by pathogenic variants (mutations) in the mismatch repair (MMR) genes which leads to an increased risk of cancers, particularly colorectal cancer (CRC) and endometrial cancer (EC) [1]. Around 1 in 300 of the general population is born with a pathogenic variant in an MMR gene (estimates include 1 in 370 [2] and 1 in 279 [3]), but these are overrepresented in cancer patients, and particularly young cancer patients.

LS is typically identified through cancer-affected individuals and testing is typically driven by tumour-based triage tests. The automatic application of these tests to eligible patients is termed *reflex testing*. When a pathogenic variant is identified, predictive testing can be offered to relatives, usually through cascade testing to minimise the number of wasted tests.

Interventions to reduce the risk of cancer incidence and mortality for families with LS include surveillance, risk-reducing surgery, and aspirin chemoprevention [1].

Reflex testing for LS is cost-effective in CRC [4–6] and is recommended in England [7]. The clinical and economic value of reflex testing for LS in EC patients may differ from reflex testing in CRC patients because: EC may be a common sentinel cancer with good survival (patients have more potential to benefit from subsequent interventions); the distribution of variants implicated in EC differs from CRC; the biomarker *BRAF* V600E, useful in CRC, has no utility in EC.

We know of three economic evaluations of reflex testing for LS in women with EC, none in the NHS setting, and none including a no testing comparator [8–10].

The aim of this study is to identify the relative cost-effectiveness of reflex testing for LS in women with EC in the NHS.

## Methods

This study used methods in line with the NICE reference case [11] to address the decision problem specified in **Table 1**and is reported in line with published criteria [12].

**Table 1:** Key design criteria for economic evaluation.

*Decision problem*	What is the relative cost-effectiveness of strategies to identify LS in women with EC?
*Intervention and comparators*	Reflex testing with MMR IHC (with or without *MLH1* methylation testing if MLH1 stain abnormal) followed by referral for LS diagnostic mutation testing Reflex testing with MSI (with or without *MLH1* methylation testing if MSI identified) followed by referral for LS diagnostic mutation testing Direct referral to genetic counselling for LS diagnostic mutation testing No testing for LS
*Model type*	Decision tree and Markov model implemented in Excel 2013 (Microsoft; Redmond, WA)
*Population*	Women newly diagnosed with EC (probands), and their relatives who may be reached for predictive testing if a pathogenic mutation is identified
*Setting*	Healthcare services, including costs to the NHS and personal social services but excluding other governmental or societal costs
*Time horizon*	Lifetime (until death or age 100 years)
*Costs*	Pounds sterling (£; GBP) in 2016/17 prices
*Benefits*	QALYs
*Discounting*	3.5% for costs and benefits
*Cost-effectiveness threshold*	£20 000 per QALY

**Key:** EC, endometrial cancer; GBP, Great Britain pounds (sterling); IHC, immunohistochemistry; LS, Lynch syndrome; MMR, mismatch repair; MSI, microsatellite instability; NHS, National Health Service; QALY, quality-adjusted life year

### Relevant population

Reflex testing is conducted in women newly diagnosed with EC (probands), so the population includes all women diagnosed with EC even if they do not have LS. Relatives may also subsequently be diagnosed with LS by predictive testing, so all relatives who could feasibly be diagnosed are modelled in the population, even if neither they nor the proband have LS or receive testing (maintaining a consistent population across interventions).

The prevalence of LS among probands declines with age, as do the opportunities to intervene meaningfully, therefore cost-effectiveness is sensitive to the age of probands. The model simulates a cohort of probands of a specific age (60 years in the base case) so that the impact of age on cost-effectiveness can be quantified.

The age distribution of relatives also affects cost-effectiveness, since younger relatives have a greater opportunity to benefit from interventions. In the base case analysis and scenario analyses we use a weighted average of the results considering the full distribution of relatives’ ages, while in the probabilistic and one-way sensitivity analyses relatives start the model aged 54 as this produces cost-effectiveness results closest to a weighted average of results across relatives’ ages.

### Selection of relevant interventions and comparators

Five reflex testing strategies were identified following consultation with clinical experts, plus a no testing option (representing current practice). Four strategies utilised tumour tests to triage; the fifth involved direct referral to genetic counselling for mutation testing. We did not include the use of tools to predict the risk of LS [13] as interventions in our base case analysis as these require taking good quality family history, which may not be feasible for some patients, incurs costs, and may be challenging in clinical settings. We have included them in a scenario analysis. It should be noted that in a fully incremental cost-effectiveness (as is conducted in this study), any convex combination of strategies is implicitly included. For example, if the true current practice was that 80% of hospitals did no testing and 20% of hospitals used a particular reflex testing strategy, the explicit inclusion of this would make no difference to the cost-effectiveness results (assuming the 20% sample was effectively random).

### Model structure

We modelled the following care pathways:

- Diagnostic testing in women with EC to identify those with LS;
- Genetic testing of male and female relatives of those women with LS to identify further cases of LS;
- Biennial colonoscopic surveillance for CRC for those having, or suspected of having LS.

The model focussed on CRC outcomes for patients as this has a significantly greater burden in terms of mortality for people with LS than other cancers. For comparison, in a prospective study of LS, incidence and 5-year survival figures suggest 4.5 deaths from colorectal cancer for every 1 death from ovarian cancer [14] (it should be noted that this was in the presence of colonoscopic surveillance, so more deaths may have been expected otherwise).

Three diagnostic categories were included: LS (pathogenic constitutional MMR variant identified), putative LS (PLS; no pathogenic variant identified, but tumour-based tests and family history suggestive of LS) and sporadic. Women diagnosed with (P)LS were offered interventions to reduce the incidence and mortality of CRC. Relatives of women diagnosed with LS were offered predictive mutation testing, with risk-reducing interventions offered to those with the variant. First-degree relatives of women diagnosed as PLS were invited to genetic counselling and offered biennial colonoscopic surveillance.

**Fig 1:**
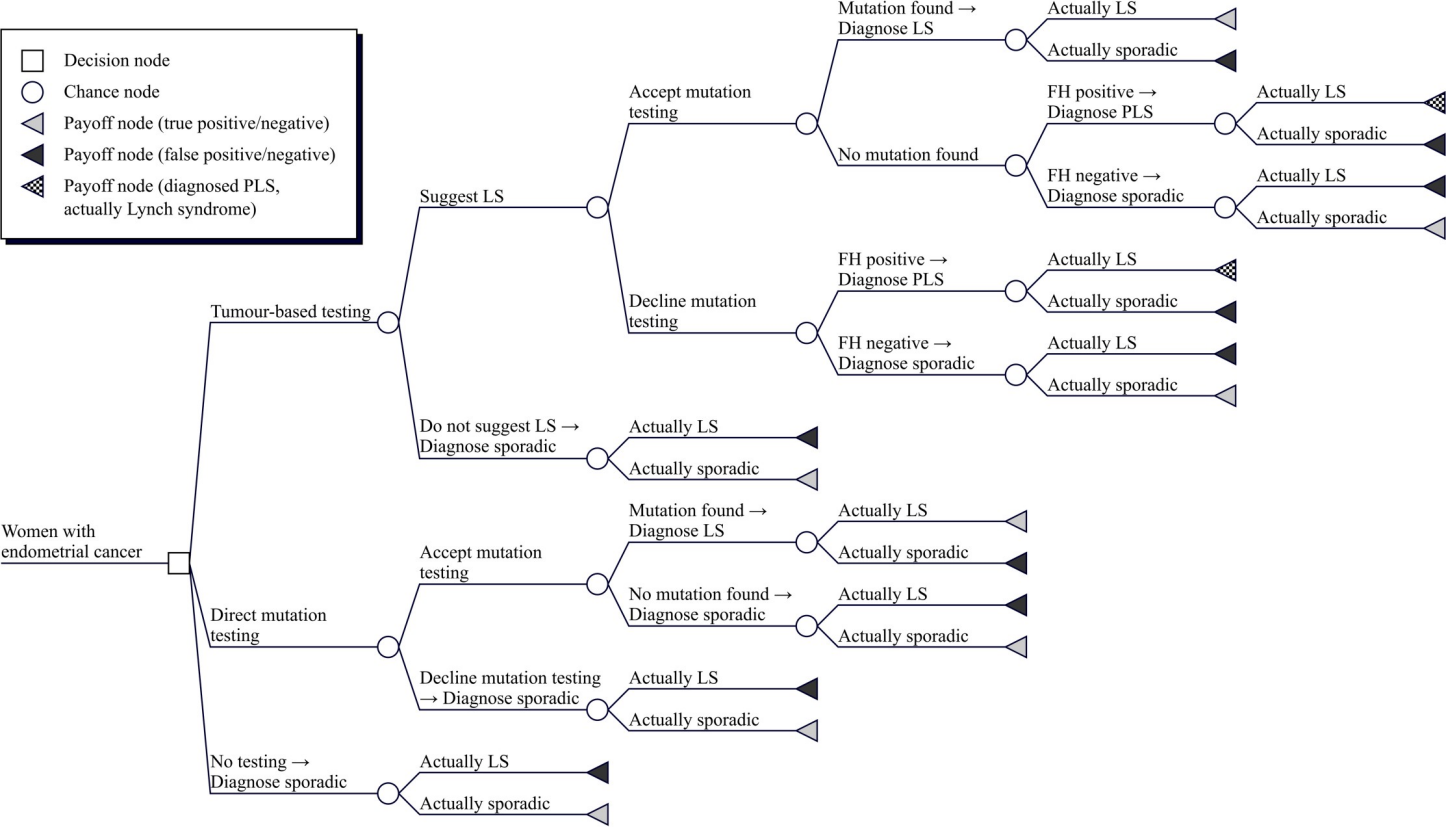
Conceptual decision tree for probands. Key: FH, family history; LS, Lynch syndrome; PLS, putative Lynch syndrome.

**Fig 2:**
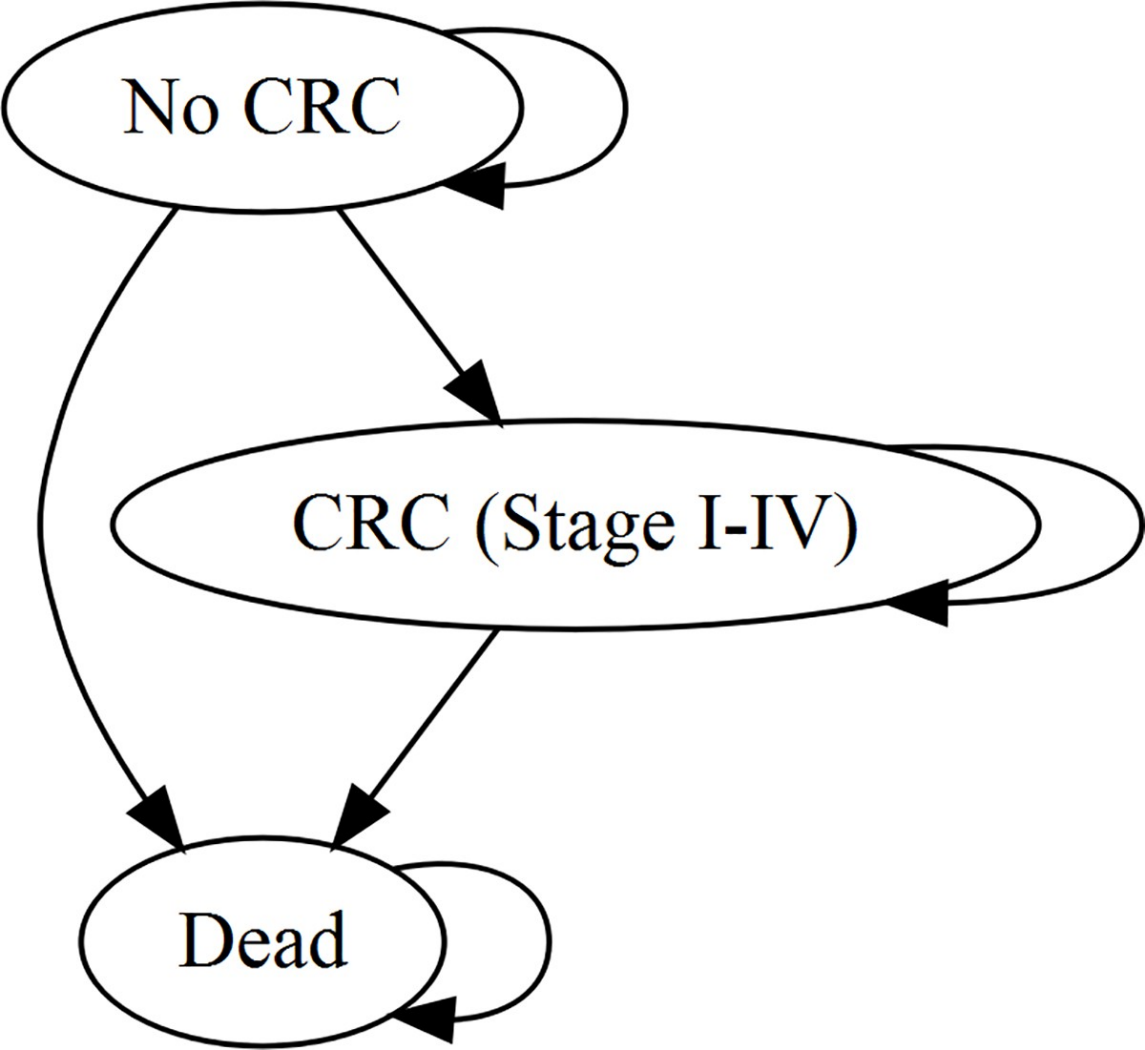
Simplified model diagram for long-term outcomes. Key: CRC, colorectal cancer. Future EC incidence was not included because all probands have already had EC and the vast majority have had a hysterectomy. Female relatives with LS are at risk of EC, but as a simplifying assumption this was not included.

The model was a decision tree (**Fig 1**) where the long-term outcomes (costs and QALYs) were estimated using a Markov model (**Fig 2**).

A Markov cycle length of one month was employed; transitions were assumed to occur midway through each cycle [15].

### Model parameters

The model inputs were populated from various sources:

- Clinical data were sourced from published literature through pragmatic literature review (**S1 Appendix**).
- Epidemiological data were sourced from published literature or national statistics.
- Cost data were sourced from published literature, NHS reference costs [16] and NHS price lists for tests as offered to other NHS providers.
- Health-related quality of life was estimated from the published literature through pragmatic literature review.

Model input parameters are described according to their function:

- Natural history–How LS is manifested in the model population in the absence of any interventions;
- Diagnostic accuracy–How accurate diagnostic tests are at detecting Lynch syndrome;
- Preventive effectiveness–How effective risk-reducing measures are;
- Utility values–The preference weights attached to different health states;
- Costs–The relevant costs identified for interventions, events and outcomes.

#### Natural history

The prevalence of LS in EC was estimated based on results from sixteen studies of testing for LS in endometrial cancer [13, 17–31] and from national statistics of six countries [32–37]. The prevalence was estimated as 3.9% overall [13, 17–31], but dependent on age and with heterogeneity between studies (**S2 Appendix**). When studies at risk of bias due to high dropout (≥ 10%) were excluded, the estimated prevalence was reduced to 3.0%.

Lynch syndrome gene mutations in *MLH1* and *MSH2* are associated with earlier age of onset of endometrial cancer than mutations in *MSH6* and *PMS2* and non-Lynch syndrome endometrial cancer. These mutations may also not be evenly distributed in the population. To estimate the distribution of gene mutations for probands of a given age, we estimated the overall relative abundance of the genes in endometrial cancer patients (of any age) by pooling studies reporting gene distributions and imposing an age limit for testing of 70 years or higher or no limit. Sixty-five endometrial cancer patients with identified MMR mutations across eight studies [18, 20–24, 28, 31] contributed to these estimates of 16.9%—24.6%—47.7%—10.8% for *MLH1—MSH2—MSH6—PMS2*. The age-specific relative abundance was then estimated by considering the age-dependent risks of endometrial cancer reported from the Prospective Lynch Syndrome Database (PLSD) [14]. A restricted cubic spline model with three knots was used, and the resulting gene distributions predicted at 5-year intervals are given in **Table 2**.

**Table 2:** MMR gene mutation distribution in endometrial cancer patients.

Age (years)	*MLH1* (%)	*MSH2* (%)	*MSH6* (%)	*PMS2* (%)
50	17.0	28.6	47.1	7.3
55	13.6	28.4	49.8	8.2
60	11.0	28.5	51.4	9.0
65	8.8	29.2	52.3	9.7
70	6.9	30.5	52.6	10.0
75	5.2	32.1	52.4	10.2
80	3.9	33.9	51.9	10.3

Probands enter the model with EC and the associated mortality risk. Survival of EC is better for women with LS compared to those with sporadic EC. The mortality rate for women with LS was estimated as 4.0 per 1000 person years based on an analysis from prospectively registered women with LS [14]. The mortality rate for sporadic EC was estimated from national statistics on 5-year survival according to age and ranged from 26.4 to 92.1 per 1000 person years [38].

To estimate the incidence of CRC in individuals with LS, survival models were fitted to reported data from the Prospective Lynch Syndrome Database (PLSD) [14, 39, 40]. Individuals with *MLH1* mutations were predicted to have the highest risk, with *MSH2*, *MSH6* and *PMS2* mutation carriers having progressively lower risks (**S3 Appendix**).

These estimates were used for individuals with LS undergoing surveillance; CRC incidence for individuals with LS not receiving surveillance was estimated by applying the (inverted) hazard ratio for incidence due to surveillance colonoscopy (see below) to these estimates.

CRC incidence for individuals without LS was estimated from national statistics [35].

CRC mortality for individuals without LS was estimated from national 5-year survival statistics [41].

CRC survival is improved for individuals with LS, except for metastatic disease. The CRC mortality rate for individuals with LS was estimated by applying a hazard ratio of 0.66 for Stage I–III CRC [42].

#### Diagnostic accuracy

For each tumour-based test, the sensitivity, specificity and test failure rate were estimated.

Meta-analyses of sensitivity and specificity were performed using the bivariate method [43] without covariates (see **S4 Appendix** for details of methods and results).

Eight studies [13, 17, 19, 21, 22, 24, 25, 30] were identified providing estimates of the diagnostic performance of IHC. The sensitivity and specificity of IHC were estimated as 94.4% and 74.8%. The test failure rate was estimated from eight studies [17, 19, 21, 22, 24, 25, 28, 31] across which 16 test failures were reported from 1037 patients, with a mean estimate of 3.7%.

The proportion of positive IHC results with absent/abnormal staining of MLH1 was assumed to be 89% in those with an *MLH1* mutation [13, 19–22, 25, 26, 30, 31, 44], 0.6% in individuals with non-*MLH1* mutations [13, 17, 19–22, 24–26, 28, 30, 31, 44], and 83% in individuals without LS [13, 17, 19–22, 24–26, 28, 30, 31, 44].

Eight studies [13, 17, 19, 21, 22, 24, 25, 30] were identified which provided estimates of the diagnostic performance of MSI. Half of these [13, 17, 25, 30] categorised tumours as MSI if ≥1/5 markers showed instability, while the rest [19, 21, 22, 24] categorised tumours as MSI if ≥2/5 markers showed instability. No threshold effect was observed, so studies were pooled without a covariate in the meta-analysis. One study [30] showed very poor sensitivity and contributed to numerical issues in the meta-analysis, and was excluded on this basis.

The sensitivity and specificity of MSI were estimated as 90.3% and 77.1%. The test failure rate was estimated from six studies [13, 19, 21, 22, 24, 25] across which 12 failures were reported from 1195 patients, with failure rates ranging from 0% to 5%, and a mean estimate of 1.9%.

Seven studies [17, 20, 21, 24, 25, 28, 31] estimated the performance of *MLH1* methylation testing following abnormal IHC results for MLH1. As no studies discovered methylated tumours with germline mutations, it is not possible to estimate the sensitivity through meta-analysis. A number of studies did not test for germline mutations if *MLH1* methylation was identified in the tumour, but two did [20, 25], and in another study [21] blood DNA was also tested to identify constitutional methylation. Sensitivity was assumed to be 95%, i.e., 95% of those with constitutional *MLH1* mutations do not show methylation. Specificity was estimated as 93.6%, applying to those without MMR mutations and those with *MSH2*, *MSH6* and *PMS2* mutations.

Only one study estimated the performance of *MLH1* methylation testing following detection of MSI [23], since all other studies conducting MSI and *MLH1* methylation testing also performed IHC and only performed *MLH1* methylation testing if MLH1 staining was absent/abnormal. Therefore, we assumed for patients with an *MLH1* mutation the sensitivity would be equivalent in patients with MSI as those with dMLH1 (95%). For patients with mutations in *MSH2*, *MSH6* and *PMS2*, we estimated that 1/14 would demonstrate *MLH1* methylation and be inappropriately discharged from further testing [20]. For patients without a constitutional MMR mutation, we assumed 67% would demonstrate *MLH1* methylation and be discharged from further testing [20]. In a scenario analyses we use estimates from Hampel et al. [23] for these parameters.

Mutations causing LS are numerous and heterogeneous. Furthermore, heritable mutations can have epigenetic effects on the MMR genes (e.g., constitutional *MLH1* methylation). Most of these LS-causing mutations are readily detected, although some may be more challenging to identify. Interpretation is coordinated internationally [45].

It was assumed diagnostic mutation testing only identifies and classifies mutations truly causing LS as pathogenic (i.e., 100% specificity). It is further assumed the sensitivity of diagnostic mutation testing is 90%, i.e., 10% of LS-causing mutations are not identified.

It was assumed predictive mutation testing is 100% accurate.

It was assumed 55% of EC patients with tumour-based test results suggestive of LS would attend genetic counselling [46], and 10% of these would decline diagnostic mutation testing [47].

#### Preventive effectiveness

The effectiveness of colonoscopic surveillance is a key determinant of the cost-effectiveness of strategies to identify LS [4, 5].

Ladabaum et al. [48] reviewed the literature and found five observational studies [49–53] estimating the impact of surveillance on CRC incidence, stage on diagnosis and mortality.

Estimates from a study by Järvinen et al. [51] have been used in a number of economic evaluations [4, 5, 47, 54]. The use of this study was considered carefully [4] in light of the higher than expected incidence of CRC in the PLSD [14], as well as the absence of evidence that more frequent colonoscopy in *MLH1* cases is more effective [55]. Of the studies identified by Ladabaum et al. [48], the study by Järvinen et al. estimates the second smallest impact on CRC incidence, with only Arrigoni et al. [49] estimating a smaller impact.

Estimates for the impact of colonoscopic surveillance on CRC incidence were therefore estimated from Järvinen et al. [5, 51] in the base case, probabilistic sensitivity analysis and one-way sensitivity analyses; and from Arrigoni et al. [49] in a scenario analysis.

CRCs were assumed to be detected in earlier stages in those undergoing surveillance [56].

#### Utility values

Consistent with the NICE reference case [11], we sought to only include direct health effects on patients. We therefore excluded non-health effects (such as information and empowerment) for which people may be prepared to forgo other consumption, and spill-over effects on family members not involved in the modelled care pathways [57, 58].

Baseline utility values (according to age and sex) were estimated from population norms [59], with impacts on health-related quality of life acting multiplicatively on baseline utility [60].

There is little evidence that non-metastatic CRC is associated with a lower (health-related) utility than population norms [5]. Therefore, it was assumed there was no disutility associated with Stage I-III CRC. Metastatic CRC is associated with significantly worse utility compared to non-metastatic CRC [61], so we scaled utility by 0.79 for Stage IV CRC.

Although health-related quality of life may be significantly worsened in a small minority of EC patients, it was assumed that on average there would be no disutility from population norms.

Genetic testing can have a number of different effects on what would broadly be considered utility, although its impact on health-related quality of life is more commonly investigated in terms of the impact on anxiety, depression and mood [57, 62].

A pragmatic search of Embase and MEDLINE using a search filter for utility values [63] failed to identify any studies measuring health-related quality of life in patients undergoing testing for LS using generic preference-based measures.

In the base case analysis, it was assumed that there would be no direct impact on QALYs from genetic counselling or genetic testing.

#### Costs

Costs are presented in 2016/17 pounds Sterling (£; GBP), inflated using the Hospital and Community Health Services Pay and Prices Index [64] to 2015/16 and then by 1.1% to 2016/17.

IHC, MSI and methylation were estimated to cost £210, £202 and £136 [4] as averages of costs reported by genetics laboratories and personal communications.

The cost of offering counselling to a proband was estimated as £27 (15 minutes of Band 6 hospital nurse time). The cost of referral for a relative was estimated as £36 (cost of a general practitioner appointment) [64].

Pre-test genetic counselling was estimated to cost £347 and £172 for probands and relatives respectively, and post-test genetic counselling was estimated to cost £133 [65].

The cost of diagnostic mutation testing for LS was estimated as £755 (the midpoint of prices offered by two genetics laboratories offering testing in all four genes for NHS patients). The cost of predictive mutation testing was estimated as £166 (the average of all costs for predictive testing in a single MMR gene [66]).

The cost of colonoscopy was estimated from NHS reference costs [16], including diagnostic and therapeutic colonoscopies (£583). Biennial colonoscopy is recommended, but it is anticipated that due to pressures on colonoscopy services and due to missed appointments, the average interval would be approximately 2.1 years.

A one-off cost of CRC is incurred at the time of CRC incidence (dependent on the patient age and stage at diagnosis), with no further cost being accrued due to time in CRC states or at time of death from CRC.

The source of cost estimates is a report by the Economic Evaluation of Health and Social Care Interventions Policy Research Unit [67], based on a whole-disease model of CRC [68]. Lifetime costs were estimated with future costs discounted at 3.5% and were assumed to be in 2010/11 prices.

#### Summary

**S5 Appendix** provides a full listing of model input parameters.

### Analysis

Fully incremental analyses are performed throughout.

A base case deterministic analysis was conducted, holding all model input parameter values fixed at a central estimate. A probabilistic sensitivity analysis (PSA) was conducted, in which parameter values were simultaneously varied according to distributions reflecting their uncertainty [69]. One-way sensitivity analyses were conducted, in which one parameter was varied across a range while other model input parameter values were fixed at their base case value. Calculations were made using the cohort method. Scenario analyses were performed to identify the importance of certain parameter and structural assumptions.

## Results

### Base case

All testing strategies were predicted to result in net QALY gains and increased costs versus no testing.

MSI with methylation was predicted to result in the least QALY gains of the testing strategies, due to diagnosing the lowest number of people with LS or as PLS. IHC-based strategies were predicted to result in greater QALY gains than equivalent MSI-based strategies as they were more sensitive. Methylation-based strategies were predicted to result in lower QALY gains than equivalent strategies without methylation testing as they are less sensitive.

IHC with methylation was the testing strategy predicted to have the lowest total cost. IHC without methylation was predicted to have the highest total cost. The use of methylation testing substantially reduced costs.

Only IHC with methylation would be considered cost-effective at a threshold of £20 000 per QALY (**Table 3**).

**Table 3:** Cost-effectiveness results.

Strategy	Incremental QALYs vs. no testing	Incremental costs vs. no testing (£)	ICER vs. no testing (£/QALY)	Fully incremental ICER (£/QALY)
*Base case*				
MSI with methylation	34.5	545 000	15 800	Dominated
Direct mutation testing	35.1	769 000	21 900	Dominated
IHC with methylation	37.9	538 000	14 200	14 200
MSI	38.3	771 000	20 100	Extendedly dominated
IHC	40.2	826 000	20 600	129 000
*Probabilistic sensitivity analysis*				
MSI with methylation	37.6	573 000	15 200	Dominated
Direct mutation testing	38.0	767 000	20 200	Dominated
IHC with methylation	41.4	554 000	13 400	13 400
MSI	42.7	855 000	20 100	Extendedly Dominated
IHC	45.1	923 000	20 500	98 800

**Key:** ICER, incremental cost-effectiveness ratio; IHC, immunohistochemistry; MSI, microsatellite instability (testing); QALY, quality-adjusted life year.

**Notes:** Based on a population of 1000 probands and 6000 relatives (average in probabilistic sensitivity analysis); Results given to 3 significant figures.

### Exploration of heterogeneity

The age of probands was varied from 40 to 85 years (not changing the distribution of the ages of relatives). The economic values of all testing strategies strictly decreased with increasing proband age. Across this range, IHC with methylation produced the most net health benefit of the testing strategies (at a willingness to pay of £20 000 per QALY), although the incremental net health benefit of this strategy (versus no testing) became negative for probands aged 65 years and upwards.

To examine the policy impact of heterogeneity, we estimated the cost-effectiveness of testing with IHC and methylation versus no testing when the population is individuals aged up to a particular age threshold. This analysis suggests that maximum economic value is achieved when the age threshold is somewhere between 60 and 65 years, but that using an age threshold of 70 years would produce more economic value than a threshold of 50 years, and that even with an age threshold of 80 years there is still positive economic value versus no testing. This happens because testing in younger endometrial cancer patients subsidises the testing in older patients.

### Exploration of uncertainty

#### Probabilistic sensitivity analysis

**Fig 3:**
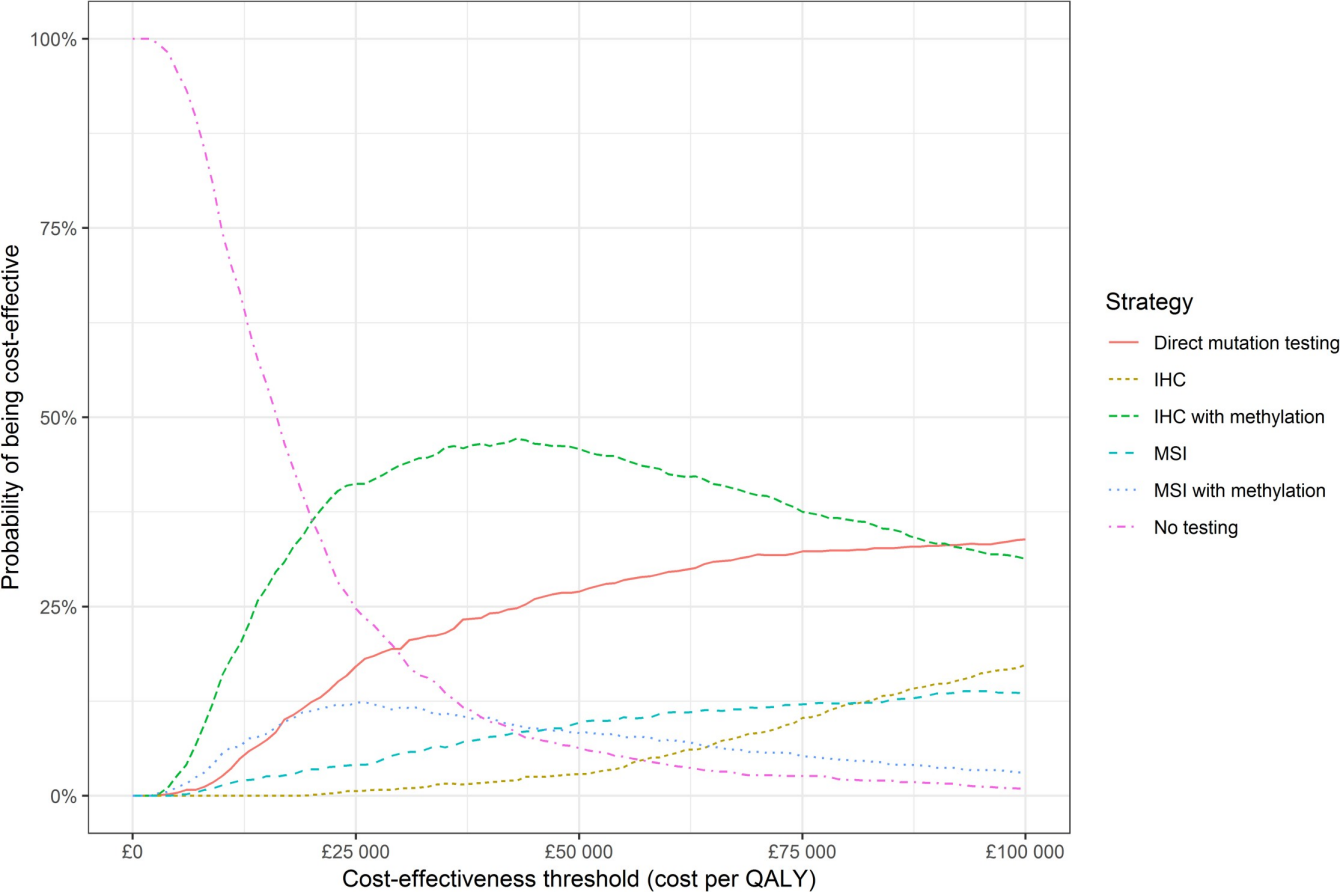
Cost-effectiveness acceptability curve from probabilistic sensitivity analysis. Key: IHC, immunohistochemistry; MSI, microsatellite instability (testing); QALY, quality-adjusted life year.

A probabilistic sensitivity analysis (PSA) was conducted with 1000 iterations. The mean results were consistent with the base case analysis, in that only IHC with methylation was predicted to be cost-effective at a threshold of £20 000 per QALY (**Table 2**). There is decision uncertainty due to parameter imprecision, since IHC with methylation is only the optimal strategy in 36% of iterations at a threshold of £20 000 per QALY (**Fig 3**). The 95% credible interval for the incremental net monetary benefit of IHC with methylation versus no testing is −£436 to £2204 per proband, and it was negative in 42% of iterations.

Another PSA was conducted with the age of probands set to 50 (instead of the base case 60 years). IHC with methylation was predicted to be the optimal strategy in 45% of iterations, and to be cost-effective compared to no testing in 90% of iterations.

#### One-way sensitivity analyses

**Fig 4:**
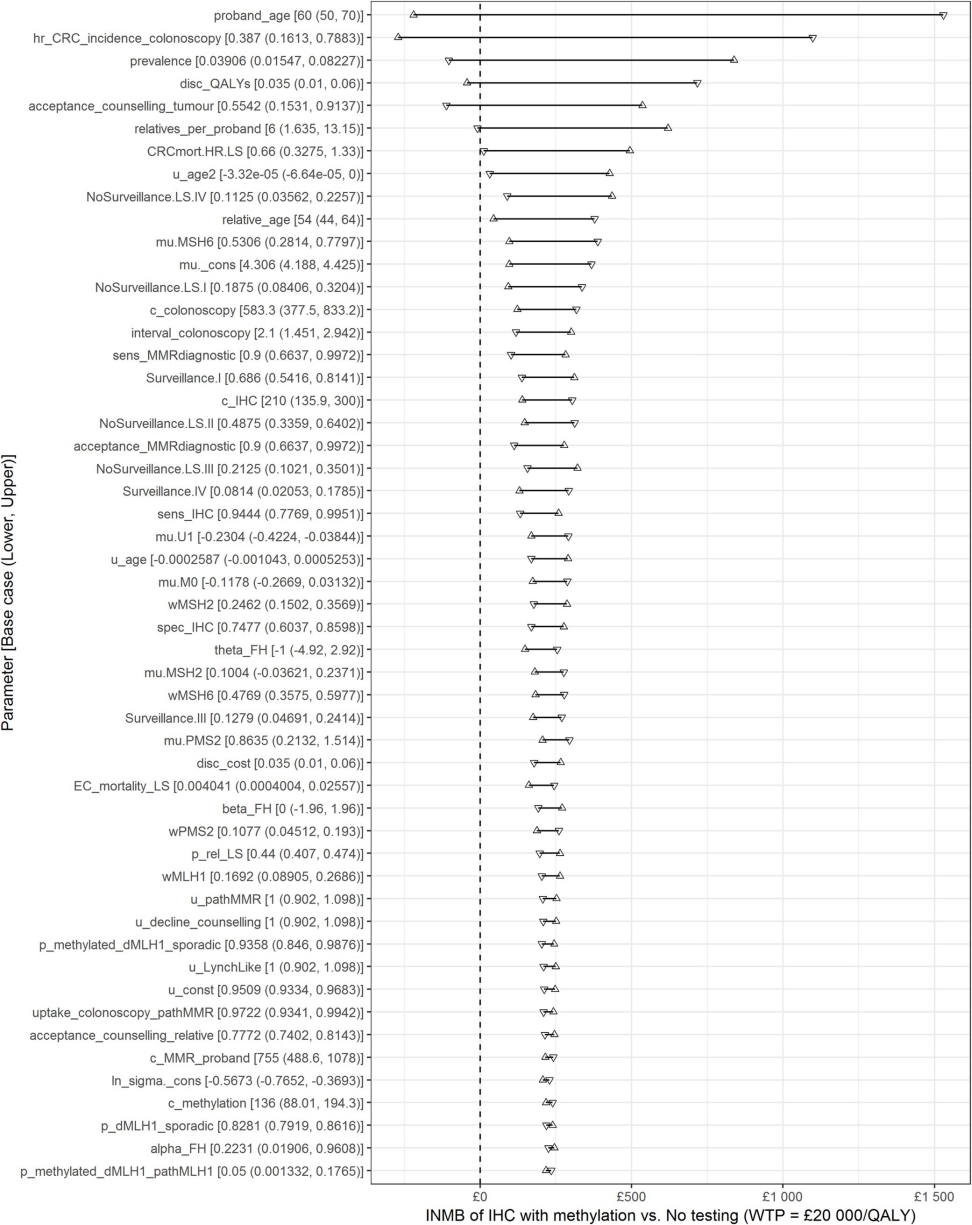
Tornado diagram of one-way sensitivity analysis. Key: IHC, immunohistochemistry; INMB, incremental net monetary benefit (presented per proband); QALY, quality-adjusted life years; WTP, willingness-to-pay; S5 Appendix gives details of all parameter abbreviations. Notes: Only includes parameters with INMB range ≥ 1% of maximum INMB range.

One-way sensitivity analyses were conducted with lower and upper parameter values equal to the 95% confidence limits for each parameter in the probabilistic sensitivity analysis (**Fig 4**). The most influential parameters are the age of the proband and the effectiveness of colonoscopy in reducing CRC incidence. Six parameters had the potential to make testing not cost-effective within the ranges considered.

#### Scenario analyses

A number of scenario analyses were conducted to examine key parameter and structural assumptions. In all but one of these scenario analyses, IHC with *MLH1* methylation testing remained cost-effective. In the scenario where the effectiveness of colonoscopic surveillance at reducing CRC incidence was estimated from Arrigoni et al. 2005 [49], none of the testing strategies was cost-effective (**S6 Appendix**). When risk prediction tools were included as testing options, these were all less effective (produced fewer QALYs) than the existing testing strategies, and they were all dominated or extendedly dominated and so would not be considered cost-effective.

## Discussion

This economic evaluation found that testing for LS in EC patients may be cost-effective in women aged 60 using MMR immunohistochemistry and *MLH1* methylation testing. Stronger conclusions cannot be drawn from the current analysis as there remains a substantial possibility that testing (especially at ages close to 60) is less cost-effective than no testing. At a lower age limit (50 years), there can be reasonable confidence that testing with IHC (or MSI) with methylation testing is cost-effective compared to no testing. Of interest, however, is the finding that the optimal programme (from a cost-effectiveness perspective) would test all endometrial cancer patients up to an age threshold somewhere between 60 and 65. Furthermore, programmes testing all endometrial cancer patients up to age 70 or 80 years would also be cost-effective compared to no testing, even though they would not be cost-effective compared to a programme using an optimised age threshold.

Three studies have previously addressed the cost-effectiveness of testing for LS in EC. Goverde et al. [8] conducted an economic evaluation alongside a trial testing for LS in 179 EC patients, extrapolating effectiveness based on previous economic evaluations. They found that testing EC <70 years with IHC and *MLH1* methylation testing would be cost-effective compared to testing EC <50 years or using the revised Bethesda guidelines. Kwon et al. [9] used a Markov model to estimate the cost-effectiveness of testing for LS in EC patients and found that using family history and IHC would be cost-effective versus using age criteria, reflex IHC or family history criteria alone. Resnick et al. [10] used a decision tree to answer a similar question, and found that using IHC could be cost-effective versus using Amsterdam criteria, depending on the value of detecting an LS case.

Our study adds significantly to the literature: it provides an estimate of cost-effectiveness in the UK setting, it includes a no testing option (important given that current practice in most settings is no testing) and measures health effects in QALYs, as opposed to life years gained or cases detected.

Our study has limitations; only CRC has been included as a downstream cancer, and we have not included risk-reducing measures besides colonoscopy. Aspirin is cheap and effective at lowering the risk of LS-associated cancers [70] and prophylactic gynaecological surgery virtually eliminates the risk of EC and ovarian cancer [71]. On the other hand, gynaecological surveillance is often recommended in spite of no convincing evidence for effectiveness or cost-effectiveness. Estimates for key parameters have also been derived through pragmatic literature review, so a systematic review, particularly one which focused on the comparative accuracy of IHC and MSI, could be valuable. Our model assumes the proportion of EC with abnormal IHC which specifically show MLH1 abnormalities, is independent of age, although some evidence suggests an association [20].

Our study also assumes that there is no testing for MMR deficiency in endometrial cancer in the no testing comparator. It may be that this testing is already being done, or will be done in the near future, to select patients for targeted immune therapies [72]. In this instance we would expect the cost-effectiveness of tumour testing-based strategies to be significantly improved for those patients as there would be zero incremental costs for those tests.

IHC is conducted to a high standard in the UK, with use of external quality assurance. It is likely that published studies based in research centres will have similarly high standards, but routine clinical settings outside the UK may have lower standards. We have also assumed (based on the literature) that IHC would use all four MMR proteins implicated in LS, although it may be argued that testing for MSH6 and PMS2 expression could be cheaper and equally effective. If a 2-protein IHC panel were equally effective and 35% cheaper than a 4-protein panel, the ICER would reduce to £12,100 per QALY compared to no testing.

We have not included any genetic testing for somatic MMR mutations, which is sometimes used (typically in research settings) to confirm that a MMR deficient tumour with no constitutional pathogenic variant identified has arisen due to somatic MMR mutations rather than from Lynch syndrome. This would be expected to increase costs. An alternative strategy for testing could include testing for pathogenic MMR variants in tumour and normal colon tissue and only referring to clinical genetics when the results suggest a constitutional pathogenic variant (which would then be confirmed by further testing). If this testing could be performed for substantially less than the cost of genetic counselling it could be cost-effective, but current estimates do not support this being the case.

There is a need for high-quality estimates of the effectiveness of surveillance colonoscopies. Although the use of randomised controlled trials is unsuitable, this does not preclude other study designs. As colonoscopies have cost implications for health services, often lead to discomfort and occasionally serious complications for those receiving them, it is important their true value in this setting is ascertained. We recommend research into the uptake of genetic counselling and testing in the UK in the context of reflex tumour-based testing. The current study assumes nearly half of patients decline genetic counselling, but this is based on a study in an insurance-based healthcare system, and could lead to an underestimate of the cost-effectiveness of testing. As shown in the one-way sensitivity analysis, if only 9% decline counselling following tumour testing, the incremental net benefit of testing is more than doubled (the ICER drops to £11,500 per QALY).

## Conclusions

We recommend that concerns of cost-effectiveness should not be a barrier to implementing reflex testing for LS in young women with EC. We recommend that the use of an age limit is justified as older women (and their relatives) have less potential to benefit from testing, as the likelihood of a positive test result is lower. Where the use of age limit is unacceptable, further research should be conducted into the cost-effectiveness of testing before it is performed in all EC patients.

## Supporting information

S1 Appendix Pragmatic literature review details. (DOCX)

S2 Appendix Estimation of the prevalence of Lynch syndrome in women with endometrial cancer. (DOCX)

S3 Appendix Estimation of the incidence of colorectal cancer in individuals with Lynch syndrome. (DOCX)

S4 Appendix Estimation of the diagnostic performance of tumour-based tests for Lynch syndrome. (DOCX)

S5 Appendix Summary of model input parameters. (DOCX)

S6 Appendix Scenario analyses. (DOCX)

S7 Appendix Estimation of life expectancy according to age and other risk factors. (DOCX)

## Abbreviations

CRC: colorectal cancer
EC: endometrial cancer
ICER: incremental cost-effectiveness ratio
IHC: immunohistochemistry
LS: Lynch syndrome
MMR: mismatch repair
MSI: microsatellite instability
NICE: National Institute for Health and Care Excellence
PLS: putative Lynch syndrome
PSA: probabilistic sensitivity analysis
QALY: quality-adjusted life year
