# Supplementary material for: Cost-effectiveness analysis of reflex testing for Lynch syndrome in women with endometrial cancer in the UK setting
Source: PLoS One. 2019 Aug 30;14(8):e0221419. doi: 10.1371/journal.pone.0221419 (PMC6716649; doi:10.1371/journal.pone.0221419)
Supplement: S1 Appendix — (DOCX) [file pone.0221419.s001.docx]

# S1 Appendix. Pragmatic literature review details

## Methods

A pragmatic literature review was conducted to identify studies of population testing for Lynch syndrome in women with endometrial cancer. Search methods included simple searches on multiple platforms (Google Scholar and Ovid [MEDLINE and EMBASE]), examining the reference lists of key papers (including reviews such as Stewart 2013 [[1](#_ENREF_1)] and Aguirre et al. 2016 [[2](#_ENREF_2)]) and forward citation chasing on identified studies (via Scopus).

Studies were included if they conducted testing for Lynch syndrome in unselected endometrial cancer patients, or patients selected only by an age limit. Consecutive patients or random sampling were both acceptable. Studies based around referrals to genetics services or using family history criteria were not included. Studies needed to conduct constitutional mutation analysis (for at least *MLH1*, *MSH2* and *MSH6*) for all patients where Lynch syndrome has not reasonably been ruled out (e.g., MSS and normal expression on four-protein MMR IHC panel, or if *MLH1* promoter hypermethylation is identified) and where patients consented. Studies needed to use at least one of MMR IHC, MSI and *MLH1* promoter hypermethylation testing.

## Results

A total of 16 studies were identified which could contribute to estimates for model input parameters, as shown in Table 1.

Table 1: Studies identified in pragmatic review

| Study | Prevalence of LS | Age distribution of LS-associated EC | Gene distribution of LS in EC | Diagnostic performance | | |
| --- | --- | --- | --- | --- | --- | --- |
|  |  |  |  | IHC | MSI | *MLH1* methylation (post IHC / post MSI) |
| Berends et al. 2003 [[3](#_ENREF_3)] | Y | Y | Y | Y | Y | N |
| Hampel et al. 2006 [[4](#_ENREF_4)], Mercado et al. 2012 [[5](#_ENREF_5)] | Y | Y | Y | Y | Y | N |
| Lu et al. 2007 [[6](#_ENREF_6)] | Y | N | Y | Y | Y | Y / Y |
| Leenen et al. 2012 [[7](#_ENREF_7)] | Y | Y | Y | Y | Y | Y / Y |
| Egoavil et al. 2013 [[8](#_ENREF_8)] | Y | Y | Y | Y | Y | Y / N c |
| Moline et al. 2013 [[9](#_ENREF_9)] | N | N | N | Y | N c | N c |
| Batte et al. 2014a [[10](#_ENREF_10)] | Y | Y | Y | N d | N d | N c |
| Buchanan et al. 2014 [[11](#_ENREF_11)] | Y | Y | Y | Y | N | Y / N |
| Ferguson et al. 2014 [[12](#_ENREF_12)] | Y | Y | Y | Y | Y | N / N |
| Mills et al. 2014 [[13](#_ENREF_13)] | Y | N | Y | N d | N d | N c |
| Mas-Moya et al. 2015 [[14](#_ENREF_14)] | Y b | N | Y | Y | N c | N c |
| Ring et al. 2016 [[15](#_ENREF_15)] | Y | N | Y | N | N | N c |
| Rubio et al. 2016 [[16](#_ENREF_16)] | Y | N | N | Y | Y | N |
| Anagnostopoulos et al. 2017 [[17](#_ENREF_17)] | Yes | Yes | Yes | Y | Y | Y / N c |
| Najdawi et al. 2017 [[18](#_ENREF_18)] | Yes | Yes | Yes | Y | N | Y / N |
| Watkins et al. 2017 [[19](#_ENREF_19)] | Yes | Yes | Yes | Y | N | Y / N |

Notes: a Prospective cohort only; b Although a significant number did not receive testing, these were treated as negative for Lynch syndrome as otherwise highly anomalous results were obtained; c It was not possible to construct a 2×2 table for this test, although the test was conducted in some/all patients; d Results only presented for patients without *MLH1* promoter hypermethylation

# References

1. Stewart A. Genetic Testing Strategies in Newly Diagnosed Endometrial Cancer Patients Aimed at Reducing Morbidity or Mortality from Lynch Syndrome in the Index Case or Her Relatives. PLoS Currents Evidence on Genomic Tests. 2013;Sep 16(Edition 1). doi: 10.1371/currents.eogt.b59a6e84f27c536e50db4e46aa26309c. PubMed PMID: 369903807.

2. Aguirre E, Grana B, Boudet M, Balmana J. Screening for Lynch syndrome among patients with newly diagnosed endometrial cancer: a comprehensive review. Tumori Journal Translated Name Tumori. 2015;102(6):548-54. Epub 2015/07/15. doi: 10.5301/tj.5000385. PubMed PMID: 26219573.

3. Berends MJ, Wu Y, Sijmons RH, van der Sluis T, Ek WB, Ligtenberg MJ, et al. Toward new strategies to select young endometrial cancer patients for mismatch repair gene mutation analysis. J Clin Oncol. 2003;21(23):4364-70. Epub 2003/12/04. doi: 10.1200/JCO.2003.04.094. PubMed PMID: 14645426.

4. Hampel H, Frankel W, Panescu J, Lockman J, Sotamaa K, Fix D, et al. Screening for Lynch syndrome (hereditary nonpolyposis colorectal cancer) among endometrial cancer patients. Cancer Res. 2006;66(15):7810-7. Epub 2006/08/04. doi: 10.1158/0008-5472.CAN-06-1114. PubMed PMID: 16885385.

5. Mercado RC, Hampel H, Kastrinos F, Steyerberg E, Balmana J, Stoffel E, et al. Performance of PREMM(1,2,6), MMRpredict, and MMRpro in detecting Lynch syndrome among endometrial cancer cases. Genet Med. 2012;14(7):670-80. Epub 2012/03/10. doi: 10.1038/gim.2012.18. PubMed PMID: 22402756; PubMed Central PMCID: PMC3396560.

6. Lu KH, Schorge JO, Rodabaugh KJ, Daniels MS, Sun CC, Soliman PT, et al. Prospective determination of prevalence of lynch syndrome in young women with endometrial cancer. J Clin Oncol. 2007;25(33):5158-64. Epub 2007/10/11. doi: 10.1200/JCO.2007.10.8597. PubMed PMID: 17925543.

7. Leenen CH, van Lier MG, van Doorn HC, van Leerdam ME, Kooi SG, de Waard J, et al. Prospective evaluation of molecular screening for Lynch syndrome in patients with endometrial cancer </= 70 years. Gynecol Oncol. 2012;125(2):414-20. Epub 2012/02/07. doi: 10.1016/j.ygyno.2012.01.049. PubMed PMID: 22306203.

8. Egoavil C, Alenda C, Castillejo A, Paya A, Peiro G, Sánchez-Heras A-B, et al. Prevalence of Lynch Syndrome among Patients with Newly Diagnosed Endometrial Cancers. PLOS ONE. 2013;8(11):e79737. doi: 10.1371/journal.pone.0079737.

9. Moline J, Mahdi H, Yang B, Biscotti C, Roma AA, Heald B, et al. Implementation of tumor testing for lynch syndrome in endometrial cancers at a large academic medical center. Gynecol Oncol. 2013;130(1):121-6. doi: 10.1016/j.ygyno.2013.04.022.

10. Batte BAL, Bruegl AS, Daniels MS, Ring KL, Dempsey KM, Djordjevic B, et al. Consequences of universal MSI/IHC in screening endometrial cancer patients for Lynch syndrome. Gynecol Oncol. 2014;134(2):319-25. doi: 10.1016/j.ygyno.2014.06.009.

11. Buchanan DD, Tan YY, Walsh MD, Clendenning M, Metcalf AM, Ferguson K, et al. Tumor mismatch repair immunohistochemistry and DNA MLH1 methylation testing of patients with endometrial cancer diagnosed at age younger than 60 years optimizes triage for population-level germline mismatch repair gene mutation testing. J Clin Oncol. 2014;32(2):90-100. Epub 2013/12/11. doi: 10.1200/JCO.2013.51.2129. PubMed PMID: 24323032; PubMed Central PMCID: PMC4876359.

12. Ferguson SE, Aronson M, Pollett A, Eiriksson LR, Oza AM, Gallinger S, et al. Performance characteristics of screening strategies for Lynch syndrome in unselected women with newly diagnosed endometrial cancer who have undergone universal germline mutation testing. Cancer. 2014;120(24):3932-9. doi: 10.1002/cncr.28933.

13. Mills AM, Liou S, Ford JM, Berek JS, Pai RK, Longacre TA. Lynch syndrome screening should be considered for all patients with newly diagnosed endometrial cancer. Am J Surg Pathol. 2014;38(11):1501-9. doi: 10.1097/PAS.0000000000000321.

14. Mas-Moya J, Dudley B, Brand RE, Thull D, Bahary N, Nikiforova MN, et al. Clinicopathological comparison of colorectal and endometrial carcinomas in patients with Lynch-like syndrome versus patients with Lynch syndrome. Hum Pathol. 2015;46(11):1616-25. doi: 10.1016/j.humpath.2015.06.022.

15. Ring KL, Bruegl AS, Allen BA, Elkin EP, Singh N, Hartman AR, et al. Germline multi-gene hereditary cancer panel testing in an unselected endometrial cancer cohort. Mod Pathol. 2016;29(11):1381-9. doi: 10.1038/modpathol.2016.135.

16. Rubio I, Ibáñez-Feijoo E, Andrés L, Aguirre E, Balmaña J, Blay P, et al. Analysis of lynch syndrome mismatch repair genes in women with endometrial cancer. Oncology. 2016;91(3):171-6. doi: 10.1159/000447972.

17. Anagnostopoulos A, McKay VH, Cooper I, Campbell F, Greenhalgh L, Kirwan J. Identifying lynch syndrome in women presenting with endometrial carcinoma under the age of 50 years. Int J Gynecol Cancer. 2017;27(5):931-7. doi: 10.1097/IGC.0000000000000962.

18. Najdawi F, Crook A, Maidens J, McEvoy C, Fellowes A, Pickett J, et al. Lessons learnt from implementation of a Lynch syndrome screening program for patients with gynaecological malignancy. Pathology. 2017;49(5):457-64. doi: 10.1016/j.pathol.2017.05.004.

19. Watkins JC, Yang EJ, Muto MG, Feltmate CM, Berkowitz RS, Horowitz NS, et al. Universal screening for mismatch-repair deficiency in endometrial cancers to identify patients with lynch syndrome and lynch-like syndrome. Int J Gynecol Pathol. 2017;36(2):115-27. doi: 10.1097/PGP.0000000000000312.
