# Supplementary material for: Cost-effectiveness analysis of reflex testing for Lynch syndrome in women with endometrial cancer in the UK setting
Source: PLoS One. 2019 Aug 30;14(8):e0221419. doi: 10.1371/journal.pone.0221419 (PMC6716649; doi:10.1371/journal.pone.0221419)
Supplement: S2 Appendix — (DOCX) [file pone.0221419.s002.docx]

# S2 Appendix. Estimation of the prevalence of Lynch syndrome in women with endometrial cancer

## Background

The prevalence of Lynch syndrome (LS) in women with endometrial cancer (EC), i.e., the probability that a given woman with EC will have LS, is dependent on age, since LS predisposes towards higher EC risk at younger ages than EC is usually observed in the general population. This means also that any study of universal testing will find a prevalence of LS dependent on whether an upper age limit is applied, and therefore any estimate of the overall prevalence of LS based on multiple studies with different age limits must account for this dependency.

## Methods

Studies were identified which reported the age at which EC patients were diagnosed with LS following some form of universal testing, as well as studies which reported the number of EC patients diagnosed with LS following universal testing. The age cut-offs for the studies were noted and were assumed to be 100 years if no age cut-off was reported.

The underlying mathematical model employed is based on Bayes’ theorem:

$$\Pr\left( LS \mid\text{EC at age ≤ }x \right)=\frac{\Pr\left( \text{EC at age ≤ }x \mid LS,EC \right)\Pr\left( LS \mid EC \right)}{\Pr\left( \text{EC at age ≤ }x \mid EC \right)}$$

$$\Pr\left( \text{EC at age }x \mid LS,EC \right)\mathcal{\sim N}\left( \mu_{LS},\sigma_{LS} \right)$$

$$\Pr\left( \text{EC at age }x \mid EC \right)\mathcal{\sim N}\left( \mu_{all},\sigma_{all} \right)$$

Studies reporting the apparent prevalence of LS provide data for $\Pr\left( LS \mid\text{EC at age ≤ }x \right)$, while studies reporting ages at which individuals are diagnosed with LS provide data for $\Pr\left( \text{EC at age ≤ }x \mid LS,EC \right)$ and national statistics provide data for $\Pr\left( \text{EC at age ≤ }x \mid EC \right)$.

Given these data, it is possible to estimate $\Pr\left( LS \mid EC \right)$ within each study, and to meta-analyse across studies using random effects (logarithmic scale).

### Age distributions with and without Lynch syndrome

To estimate the age distribution at which women with LS develop EC (without any adjustment for underlying demographics), truncated regression with robust variance estimation (treating each study as a cluster) was employed.

The reference age distribution at which women would be expected to develop EC in each study (without any adjustment for underlying demographics) was estimated by interval regression using published national statistics [[1-6](#_ENREF_1)]. The estimates from these national statistics were then combined using the normal approximation for a mixture of normal distributions:

$$\mu=\sum_{i} w_{i}\mu_{i}$$

$$\sigma^{2}=\sum_{i} w_{i}\left( \left( \mu_{i}-\mu\right)^{2}+\sigma_{i}^{2} \right)$$

### Within study estimation of prevalence of Lynch syndrome in endometrial cancer patients

In a significant number of studies, not all women with endometrial cancer whose tumour-based tests were suggestive of Lynch syndrome went on to receive genetic testing, for reasons such as choosing not to undergo counselling or testing, or being lost to follow-up.

The prevalence of Lynch syndrome in each study was therefore estimated using the following statistic:

$$\hat{p}_{i}=\text{Prevalence in study }i=\frac{\text{Triage positive}}{\text{Triage tested}}\times\frac{\text{MMR positive}}{\text{MMR tested}}=\frac{k_{i1}}{n_{i1}}\times\frac{k_{i2}}{n_{i2}}=\hat{p}_{i1}\hat{p}_{i2}$$

This was estimated on a log-scale:

$$\ln\hat{p}_{ij}=\ln k_{ij}-\ln n_{ij}$$

$$\mathrm{Var} \left[ \ln\hat{p}_{ij} \right]=\frac{1-\hat{p}_{ij}}{n_{ij}\hat{p}_{ij}}$$

$$\ln\hat{p}_{i}=\ln\hat{p}_{i1}+\ln\hat{p}_{i2}$$

$$\mathrm{Var} \left[ \ln\hat{p}_{i} \right]=\mathrm{Var} \left[ \ln\hat{p}_{i1} \right]+\mathrm{Var} \left[ \ln\hat{p}_{i2} \right]$$

Note that this calculation assumes that dropout between those who triage positive and get MMR mutation testing is independent of the outcome of MMR testing. This would be violated if, for example, people were more likely to be tested if they had a significant family history.

### Synthesis across studies

A random effects meta-analysis was conducted to estimate the overall prevalence of Lynch syndrome among women with endometrial cancer, $p$.

A maximum likelihood approach was utilised, with each study having the following log-likelihood contribution:

$$\ln L_{i}=\frac{\left( \hat{\theta}_{i}-\theta_{i}\left( \hat{p} \right) \right)^{2}}{2\left( s_{i}^{2}+\tau^{2} \right)}-\frac{1}{2}\ln\left( 2\pi\left( s_{i}^{2}+\tau^{2} \right) \right)$$

In which $\hat{\theta}_{i}=\ln\hat{p}_{i}$, $s_{i}^{2}=\mathrm{Var} \left[ \ln\hat{p}_{i} \right]$, $\tau^{2}$ is the random effects (between-study) variance and $\theta_{i}\left( \hat{p} \right)$ is the modelled log-prevalence for study $i$:

$$\theta_{i}\left( \hat{p} \right)=\ln\left( \frac{\Phi\left( \frac{x_{i}-\mu_{LS}}{\sigma_{LS}} \right)}{\Phi\left( \frac{x_{i}-\mu_{LS}}{\sigma_{LS}} \right)\hat{p}+\Phi\left( \frac{x_{i}-\mu_{noLS}}{\sigma_{noLS}} \right)\left( 1-\hat{p} \right)} \right)$$

And $x_{i}$ is the upper age limit used in study $i$.

## Results

### Age distributions with and without Lynch syndrome

Ten studies were identified providing the ages of individual women diagnosed with EC and subsequently diagnosed with LS by some form of universal testing. These studies accounted for 77 women with LS subsequently diagnosed with LS from six countries.

Table 1: Age distributions within and across countries

| Country | Patients (studies) | Age distribution with Lynch syndrome a, b | Age distribution in country |
| --- | --- | --- | --- |
| Australia | 29 (2) | 52.2 ± 8.9 | 64.9 ± 12.3 |
| USA | 18 (3) | 54.1 ± 10.3 | 62.8 ± 12.2 |
| Netherlands | 12 (2) | 52.4 ± 9.0 | 68.6 ± 10.7 |
| Spain | 8 (1) | 49.0 ± 8.6 | 66.4 ± 12.0 |
| Canada | 7 (1) | 44.1 ± 9.8 | 63.8 ± 11.6 |
| UK | 3 (1) | 47.3 ± 1.2 | 67.4 ± 11.6 |
| Combined | 77 (10) | 52.4 ± 9.3 | 65.2 ± 12.1 |

Notes: a For each individual country this is simply the mean and standard deviation, with no adjustment for truncation; b The combined estimate includes adjustment for truncation and clustering within studies

### Estimates of prevalence by study

Table 2 provides the data and prevalence estimates from each of the studies. As shown in Figure 1, it appears that the estimated prevalence may be upwardly biased in studies with high dropout (suggesting that progression to MMR testing is non-random but is related to the likelihood of finding a mutation).

Table 2: Estimates of prevalence within studies

| Study | Triage | | MMR | | Prevalence [%] | |
| --- | --- | --- | --- | --- | --- | --- |
|  | Tested | Positive | Tested | Positive | Point | 95% CI |
| Age limit 50 |  |  |  |  |  |  |
| Lu 2007 [[7](#_ENREF_7)] | 100 | 33 | 33 | 9 | 9.0 | 4.8–16.8 |
| Berends 2003 [[8](#_ENREF_8)] | 58 | 30 | 30 | 5 | 8.6 | 3.7–19.9 |
| Anagnostopoulos 2017 [[9](#_ENREF_9)] | 35 | 11 | 9 | 3 | 10.5 | 3.7–29.8 |
| Rubio 2016 [[10](#_ENREF_10)] |  |  | 103 | 8 | 7.8 | 4.0–15.1 |
| Age limit 70 |  |  |  |  |  |  |
| Leenen 2012 [[11](#_ENREF_11)] | 183 | 11 | 10 | 7 | 4.2 | 2.1–8.5 |
| Age limit 80 |  |  |  |  |  |  |
| Buchanan 2014 [[12](#_ENREF_12)] | 702 | 170 | 158 | 22 | 3.4 | 2.2–5.1 |
| No age limit |  |  |  |  |  |  |
| Mercado 2012 [[13](#_ENREF_13)] | 563 | 131 | 131 | 14 | 2.5 | 1.5–4.2 |
| Mas-Moya 2015 [[14](#_ENREF_14)] | 215 | 37 | 17 | 11 | 11.1 | 7.0–17.6 |
| Mills 2014 [[15](#_ENREF_15)] | 605 | 40 | 21 | 17 | 5.4 | 3.7–7.7 |
| Najdawi 2017 [[16](#_ENREF_16)] | 124 | 11 | 9 | 3 | 3.0 | 1.0–8.7 |
| Batte 2014 [[17](#_ENREF_17)] | 206 | 18 | 10 | 4 | 3.5 | 1.5–8.4 |
| Ring 2016 [[18](#_ENREF_18)] |  |  | 381 | 22 | 5.8 | 3.8–8.7 |
| Egoavil 2013 [[19](#_ENREF_19)] | 173 | 27 | 19 | 8 | 6.6 | 3.5–12.3 |
| Ferguson 2014 [[20](#_ENREF_20)] | 118 | 20 | 16 | 7 | 7.4 | 3.7–14.7 |
| Watkins 2016 [[21](#_ENREF_21)] | 242 | 11 | 10 | 4 | 1.8 | 0.7–4.7 |


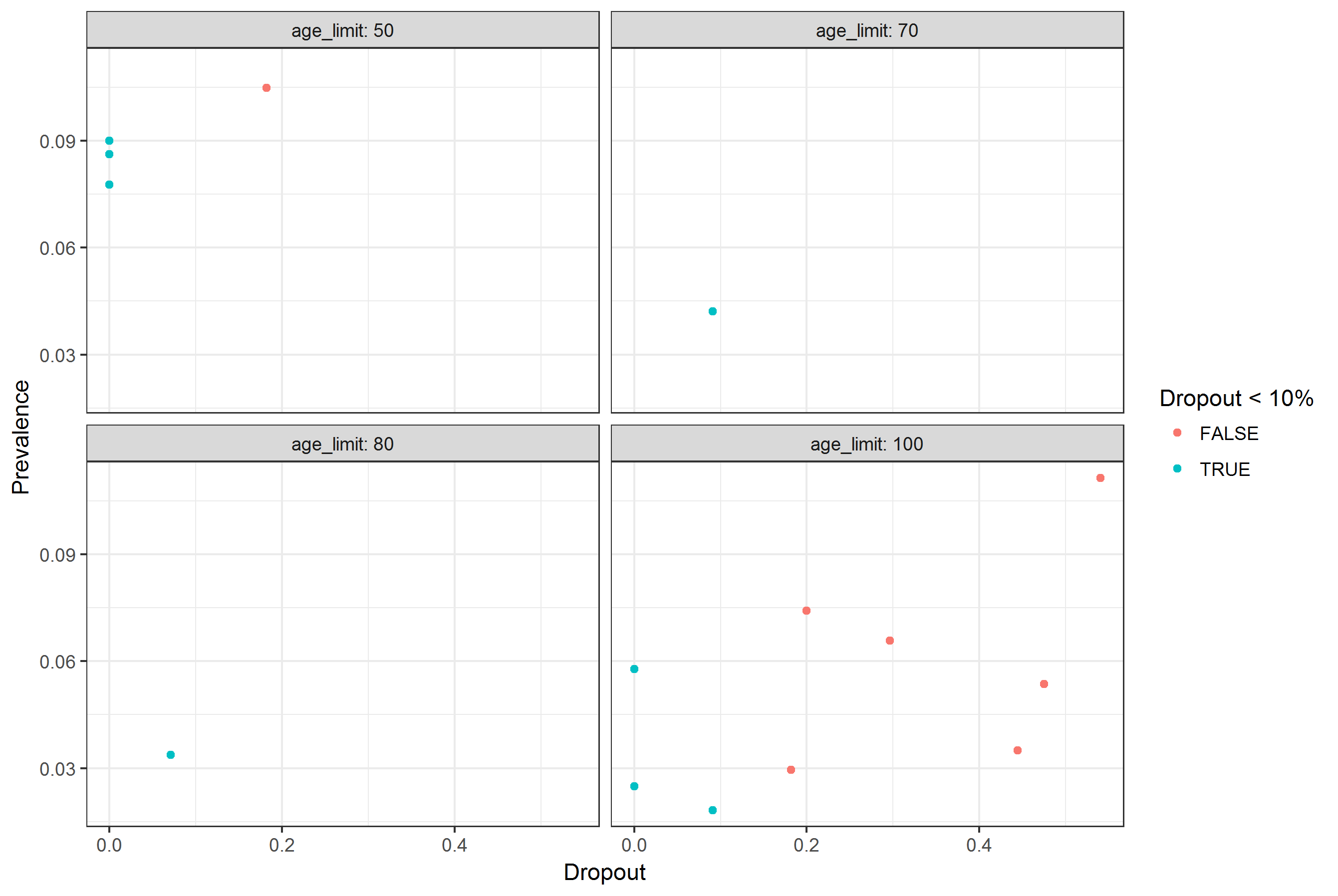


Figure 1: Estimated prevalence according to dropout rate between triage and MMR testing

### Synthesis across studies

Table 3: Random effects meta-analysis of prevalence

| Parameter | Estimate | Standard error | 95% CI |
| --- | --- | --- | --- |
| All studies (n = 15) |  |  |  |
| Prevalence (log-scale) | −3.243 | 0.142 | −3.521, −2.964 |
| Prevalence | 0.039 | 0.006 | 0.028, 0.050 |
| Random effects variance of log-prevalence (log-scale) | −0.860 | 0.280 | −1.410, −0.310 |
| Random effects variance of log-prevalence | 0.423 | 0.119 | 0.244, 0.733 |
| Low dropout studies (n = 8) |  |  |  |
| Prevalence (log-scale) | −3.513 | 0.146 | −3.799, −3.226 |
| Prevalence | 0.030 | 0.004 | 0.021, 0.039 |
| Random effects variance of log-prevalence (log-scale) | −1.406 | 0.527 | −2.438, 0.374 |
| Random effects variance of log-prevalence | 0.245 | 0.129 | 0.087, 1.646 |


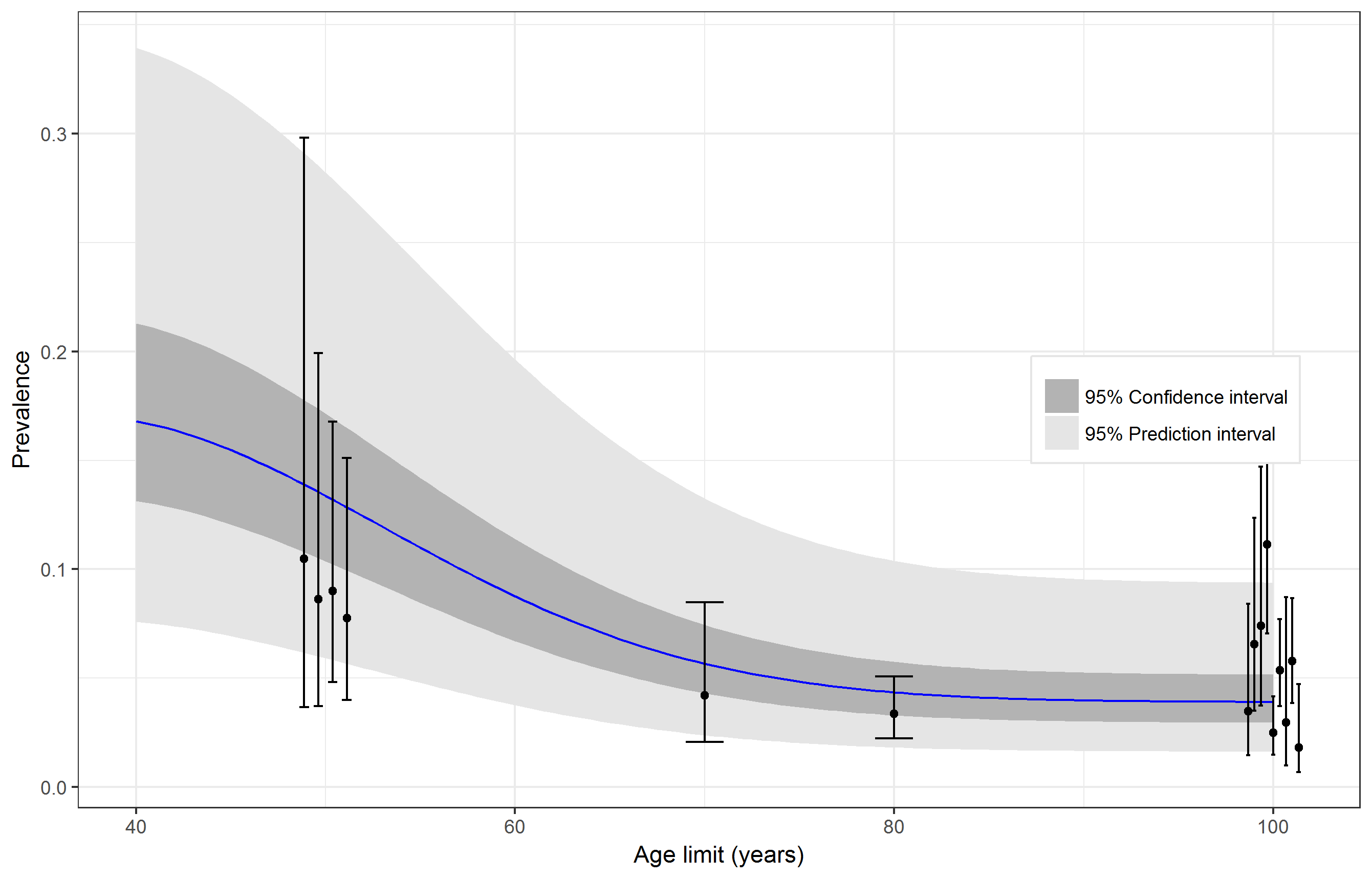


Figure 2: Fitted model for prevalence (based on all 15 studies)


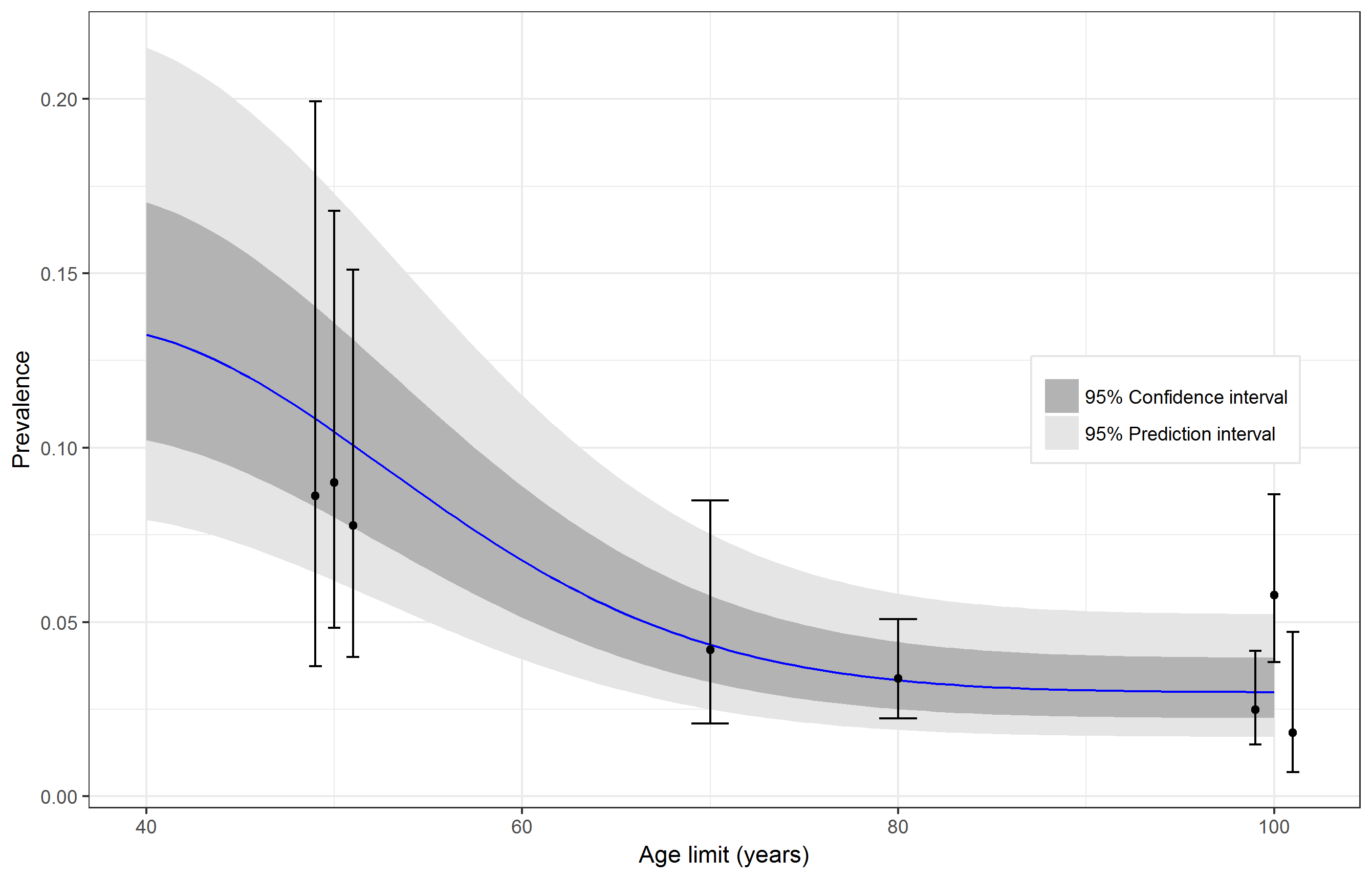


Figure 3: Fitted model for prevalence (8 studies with dropout < 10% between triage and MMR testing)

# References

1. Australian Institute of Health and Welfare. Australian Cancer Incidence and Mortality (ACIM) books 2017 [cited 2018 9 April]. Available from: <https://www.aihw.gov.au/reports/cancer/acim-books/contents/acim-books>.

2. Howlader N, Noone AM, Krapcho M, Miller D, Bishop K, Kosary CL, et al. SEER Cancer Statistics Review, 1975-2014. Bethesda, MD: National Cancer Institute; 2017.

3. Integraal Kankercentrum Nederland. The Netherlands Cancer Registry 2018 [cited 2018 9 April]. Available from: <https://www.cijfersoverkanker.nl/?language=en>.

4. Office for National Statistics. Cancer registration statistics, England: 2015 2017 [cited 2017 December 15]. Available from: <https://www.ons.gov.uk/peoplepopulationandcommunity/healthandsocialcare/conditionsanddiseases/datasets/cancerregistrationstatisticscancerregistrationstatisticsengland>.

5. Statistics Canada. Number and rates of new cases of primary cancer (CANSIM Table 103-0550) 2018 [cited 2018 9 April]. Available from: <http://www5.statcan.gc.ca/cansim/a47>.

6. Steliarova-Foucher E, O'Callaghan M, Ferlay J, Masuyer E, Forman D, Comber H, et al. European Cancer Observatory: Cancer incidence, mortality, prevalence and survival in Europe 2012 [cited 2018 9 April]. Available from: <http://eco.iarc.fr/>.

7. Lu KH, Schorge JO, Rodabaugh KJ, Daniels MS, Sun CC, Soliman PT, et al. Prospective determination of prevalence of lynch syndrome in young women with endometrial cancer. J Clin Oncol. 2007;25(33):5158-64. Epub 2007/10/11. doi: 10.1200/JCO.2007.10.8597. PubMed PMID: 17925543.

8. Berends MJ, Wu Y, Sijmons RH, van der Sluis T, Ek WB, Ligtenberg MJ, et al. Toward new strategies to select young endometrial cancer patients for mismatch repair gene mutation analysis. J Clin Oncol. 2003;21(23):4364-70. Epub 2003/12/04. doi: 10.1200/JCO.2003.04.094. PubMed PMID: 14645426.

9. Anagnostopoulos A, McKay VH, Cooper I, Campbell F, Greenhalgh L, Kirwan J. Identifying lynch syndrome in women presenting with endometrial carcinoma under the age of 50 years. Int J Gynecol Cancer. 2017;27(5):931-7. doi: 10.1097/IGC.0000000000000962.

10. Rubio I, Ibáñez-Feijoo E, Andrés L, Aguirre E, Balmaña J, Blay P, et al. Analysis of lynch syndrome mismatch repair genes in women with endometrial cancer. Oncology. 2016;91(3):171-6. doi: 10.1159/000447972.

11. Leenen CH, van Lier MG, van Doorn HC, van Leerdam ME, Kooi SG, de Waard J, et al. Prospective evaluation of molecular screening for Lynch syndrome in patients with endometrial cancer </= 70 years. Gynecol Oncol. 2012;125(2):414-20. Epub 2012/02/07. doi: 10.1016/j.ygyno.2012.01.049. PubMed PMID: 22306203.

12. Buchanan DD, Tan YY, Walsh MD, Clendenning M, Metcalf AM, Ferguson K, et al. Tumor mismatch repair immunohistochemistry and DNA MLH1 methylation testing of patients with endometrial cancer diagnosed at age younger than 60 years optimizes triage for population-level germline mismatch repair gene mutation testing. J Clin Oncol. 2014;32(2):90-100. Epub 2013/12/11. doi: 10.1200/JCO.2013.51.2129. PubMed PMID: 24323032; PubMed Central PMCID: PMC4876359.

13. Mercado RC, Hampel H, Kastrinos F, Steyerberg E, Balmana J, Stoffel E, et al. Performance of PREMM(1,2,6), MMRpredict, and MMRpro in detecting Lynch syndrome among endometrial cancer cases. Genet Med. 2012;14(7):670-80. Epub 2012/03/10. doi: 10.1038/gim.2012.18. PubMed PMID: 22402756; PubMed Central PMCID: PMC3396560.

14. Mas-Moya J, Dudley B, Brand RE, Thull D, Bahary N, Nikiforova MN, et al. Clinicopathological comparison of colorectal and endometrial carcinomas in patients with Lynch-like syndrome versus patients with Lynch syndrome. Hum Pathol. 2015;46(11):1616-25. doi: 10.1016/j.humpath.2015.06.022.

15. Mills AM, Liou S, Ford JM, Berek JS, Pai RK, Longacre TA. Lynch syndrome screening should be considered for all patients with newly diagnosed endometrial cancer. Am J Surg Pathol. 2014;38(11):1501-9. doi: 10.1097/PAS.0000000000000321.

16. Najdawi F, Crook A, Maidens J, McEvoy C, Fellowes A, Pickett J, et al. Lessons learnt from implementation of a Lynch syndrome screening program for patients with gynaecological malignancy. Pathology. 2017;49(5):457-64. doi: 10.1016/j.pathol.2017.05.004.

17. Batte BAL, Bruegl AS, Daniels MS, Ring KL, Dempsey KM, Djordjevic B, et al. Consequences of universal MSI/IHC in screening endometrial cancer patients for Lynch syndrome. Gynecol Oncol. 2014;134(2):319-25. doi: 10.1016/j.ygyno.2014.06.009.

18. Ring KL, Bruegl AS, Allen BA, Elkin EP, Singh N, Hartman AR, et al. Germline multi-gene hereditary cancer panel testing in an unselected endometrial cancer cohort. Mod Pathol. 2016;29(11):1381-9. doi: 10.1038/modpathol.2016.135.

19. Egoavil C, Alenda C, Castillejo A, Paya A, Peiro G, Sánchez-Heras A-B, et al. Prevalence of Lynch Syndrome among Patients with Newly Diagnosed Endometrial Cancers. PLOS ONE. 2013;8(11):e79737. doi: 10.1371/journal.pone.0079737.

20. Ferguson SE, Aronson M, Pollett A, Eiriksson LR, Oza AM, Gallinger S, et al. Performance characteristics of screening strategies for Lynch syndrome in unselected women with newly diagnosed endometrial cancer who have undergone universal germline mutation testing. Cancer. 2014;120(24):3932-9. doi: 10.1002/cncr.28933.

21. Watkins JC, Yang EJ, Muto MG, Feltmate CM, Berkowitz RS, Horowitz NS, et al. Universal screening for mismatch-repair deficiency in endometrial cancers to identify patients with lynch syndrome and lynch-like syndrome. Int J Gynecol Pathol. 2017;36(2):115-27. doi: 10.1097/PGP.0000000000000312.
