# Supplementary material for: Cost-effectiveness analysis of reflex testing for Lynch syndrome in women with endometrial cancer in the UK setting
Source: PLoS One. 2019 Aug 30;14(8):e0221419. doi: 10.1371/journal.pone.0221419 (PMC6716649; doi:10.1371/journal.pone.0221419)
Supplement: S3 Appendix — (DOCX) [file pone.0221419.s003.docx]

# S3 Appendix. Estimation of the incidence of colorectal cancer in individuals with Lynch syndrome

## Background

It has been historically very challenging to obtain unbiased estimates of the cancer risk in Lynch syndrome because many families with Lynch syndrome were identified specifically because they had a high incidence of cancer (ascertainment bias) [[1](#_ENREF_1)]. Different statistical methods have been employed to correct for this bias, but these typically reduce statistical power considerably.

Furthermore, many efforts have focused on establishing the risk in individuals who do not receive risk-reducing interventions, but this typically means that power is reduced further since observations after an individual’s diagnosis needs to be discarded [[1](#_ENREF_1)].

More recently, focus has shifted to estimating cancer risk for individuals who do receive risk-reducing interventions, and to collect prospective data as this is not subject to ascertainment bias (although it may still be affected by selection bias).

## Methods

To estimate the incidence of CRC in individuals with LS, according to MMR gene mutated, parametric, non-parametric and flexible spline models were fitted to reported data from the Prospective Lynch Syndrome Database (PLSD) [[2-4](#_ENREF_2)]. Data were aggregated within five-year age groups and included the number of person-years observed ($exposure$), the number of cases ($cases$), the gene mutated and (for participants without previous cancer) sex. Maximum likelihood estimates of parameters were obtained using the following model:

$$cases\sim Bin\left( \frac{exposure}{5};1-\frac{S\left( t_{u} \right)}{S\left( t_{l} \right)} \right)$$

Where $S\left( t_{u} \right)$ and $S\left( t_{l} \right)$ are the cancer-free survival estimates at the upper and lower ages for each age group, based on a given parametric model.

Models were compared using the Akaike Information Criterion.

The parametric models were all fitted with the primary parameter (e.g., $\lambda$ for Weibull, $\mu$ for lognormal) being estimated according to gene, sex and previous cancer (these last two variables were combined into a single factor with three levels as sex was not reported if a participant had previous cancer). These models all performed better (in terms of Akaike Information Criterion [AIC]) than equivalent models including only some, or none, of these covariates. There was insufficient data to estimate interactions between sex/previous cancer and gene.

Ancillary parameters (e.g., $\gamma$ for Weibull, $\sigma$ for lognormal) were estimated without covariates.

In addition to the parametric models, a Poisson model was also fitted with age group as a factor (in addition to gene, sex and previous cancer), as well as a Poisson model with a restricted cubic spline for age. The knot positions were as recommended by Harrell [[5](#_ENREF_5)] and the number of knots was selected to minimise AIC.

## Results

Table 1 summarises the performance of the different models. The lognormal distribution was found to give the best fit. The corresponding parameters are provided in Table 2 and the resulting hazard profile is shown in Figure 1. Figure 2 compares the observed number of cases in the Prospective Lynch Syndrome Database to the prediction from the lognormal model.

Table 1: Model fit statistics for colorectal cancer incidence for individuals with Lynch syndrome

| Model | Log-likelihood | Degrees of freedom | AIC |
| --- | --- | --- | --- |
| Exponential | -131.0861 | 6 | 274.1722 |
| Weibull | -124.4277 | 7 | 262.8554 |
| Gompertz | -124.886 | 7 | 263.772 |
| Lognormal | -122.9367 | 7 | 259.8734 |
| Loglogistic | -123.6334 | 7 | 261.2668 |
| Poisson | -120.4539 | 14 | 268.9077 |
| Poisson + Spline(3) | -125.0889 | 8 | 266.1778 |

Table 2: Impact of patient characteristics on colorectal cancer incidence (lognormal model)

| Characteristic | Acceleration factor | 95% confidence interval |
| --- | --- | --- |
| Gene mutated |  |  |
| *MLH1* | (Reference) |  |
| *MSH2* | 0.904 | 0.781–1.028 |
| *MSH6* | 0.588 | 0.442–0.735 |
| *PMS2* | 0.422 | 0.147–0.696 |
| Sex / Previous cancer |  |  |
| Female / No previous cancer | (Reference) |  |
| Male / No previous cancer | 1.125 | 0.957–1.293 |
| Previous cancer | 1.259 | 1.017–1.501 |


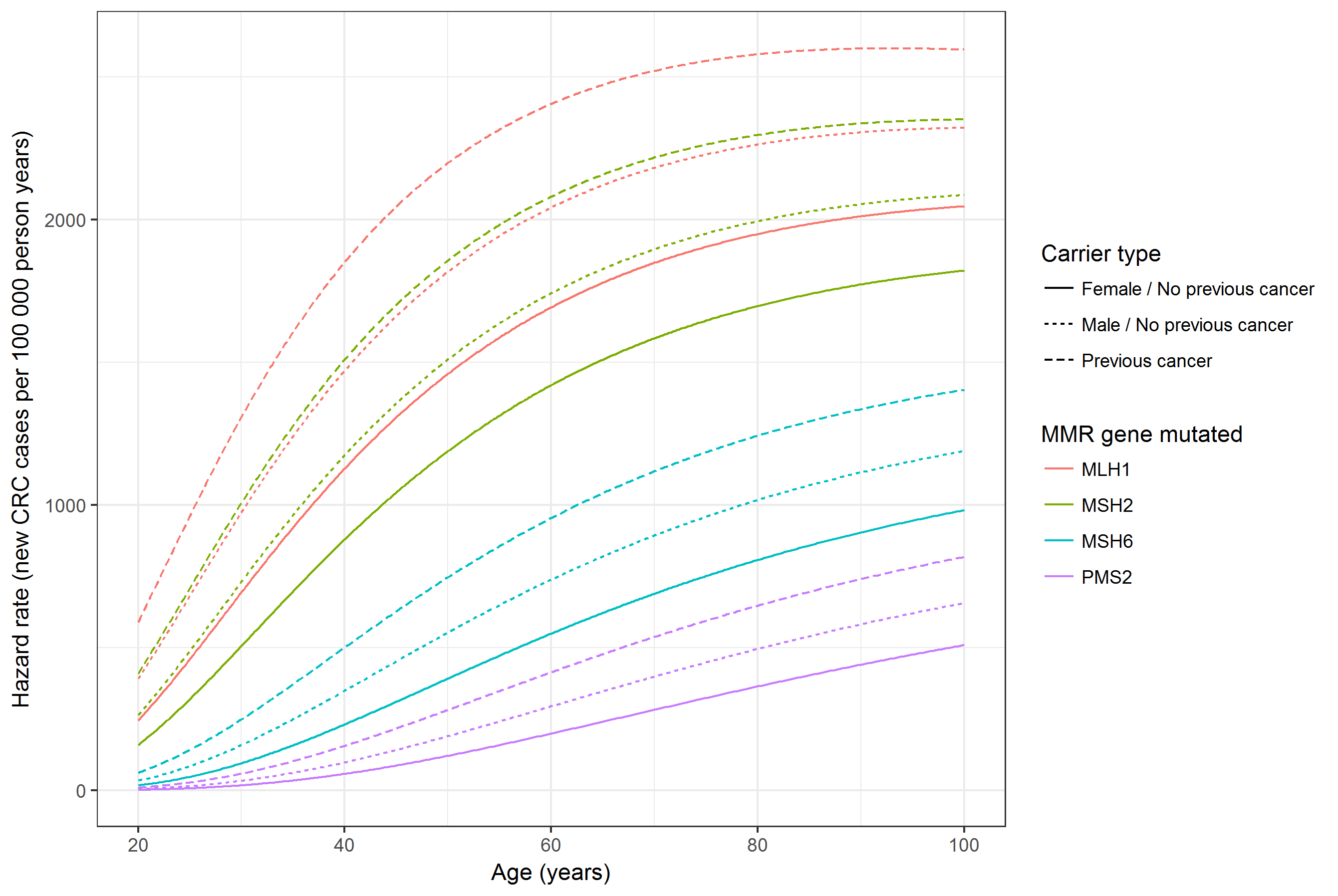


Figure 1: Hazard profile for colorectal cancer incidence (lognormal model)

Individuals with *MLH1* mutations were predicted to have the highest risk, with *MSH2*, *MSH6* and *PMS2* mutation carriers having progressively lower risks. Men were predicted to have higher risks than women among those without previous cancer. Those with previous cancer had higher predicted risks than those without previous cancer.


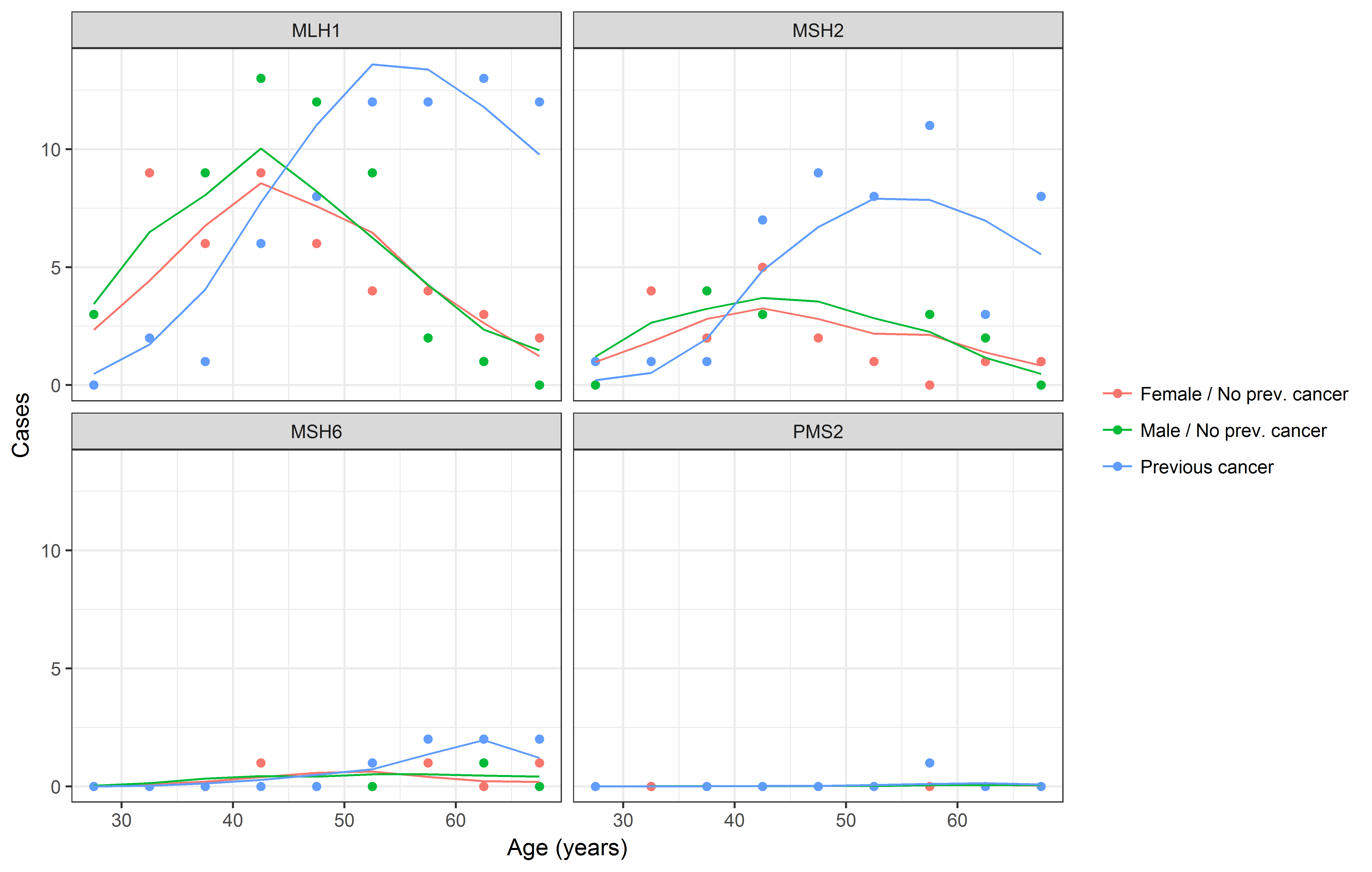


Figure 2: Comparison of observed and predicted (lognormal model) colorectal cancer cases

Key: Points represent observations from the Prospective Lynch Syndrome Database [[2-4](#_ENREF_2)] while lines represent model predictions

# References

1. Bonadona V, Bonaiti B, Olschwang S, Grandjouan S, Huiart L, Longy M, et al. Cancer risks associated with germline mutations in MLH1, MSH2, and MSH6 genes in Lynch syndrome. JAMA. 2011;305(22):2304-10. Epub 2011/06/07. doi: 10.1001/jama.2011.743. PubMed PMID: 21642682.

2. Moller P, Seppala TT, Bernstein I, Holinski-Feder E, Sala P, Evans DG, et al. Cancer risk and survival in path_MMR carriers by gene and gender up to 75 years of age: a report from the Prospective Lynch Syndrome Database. Gut. 2017. Epub 2017/07/30. doi: 10.1136/gutjnl-2017-314057. PubMed PMID: 28754778.

3. Moller P, Seppala T, Bernstein I, Holinski-Feder E, Sala P, Evans DG, et al. Incidence of and survival after subsequent cancers in carriers of pathogenic MMR variants with previous cancer: a report from the prospective Lynch syndrome database. Gut. 2017;66(9):1657-64. Epub 2016/06/05. doi: 10.1136/gutjnl-2016-311403. PubMed PMID: 27261338; PubMed Central PMCID: PMC5561364.

4. Moller P, Seppala T, Bernstein I, Holinski-Feder E, Sala P, Evans DG, et al. Cancer incidence and survival in Lynch syndrome patients receiving colonoscopic and gynaecological surveillance: first report from the prospective Lynch syndrome database. Gut. 2017;66(3):464-72. Epub 2015/12/15. doi: 10.1136/gutjnl-2015-309675. PubMed PMID: 26657901; PubMed Central PMCID: PMC5534760.

5. Harrell FE. Regression modeling strategies : with applications to linear models, logistic regression, and survival analysis. New York: Springer; 2001.
