# Supplementary material for: Cost-effectiveness analysis of reflex testing for Lynch syndrome in women with endometrial cancer in the UK setting
Source: PLoS One. 2019 Aug 30;14(8):e0221419. doi: 10.1371/journal.pone.0221419 (PMC6716649; doi:10.1371/journal.pone.0221419)
Supplement: S4 Appendix — (DOCX) [file pone.0221419.s004.docx]

# S4 Appendix. Estimation of the diagnostic performance of tumour-based tests for Lynch syndrome

## Methods

Studies were identified from the pragmatic literature review (**S1 Appendix**) from which 2×2 test accuracy tables could be calculated for MSI and IHC. Additionally, studies which included *MLH1* methylation testing were identified and classified according to whether IHC or MSI was the preceding test; however, all relevant details for these analyses are included in the main paper.

The reference standard for the 2×2 tables was as described in the studies, but unclassified variants were treated as reference standard negative (i.e., sporadic), and patients with tumour-based test results suggesting Lynch syndrome but not receiving germline testing were excluded within the analyses.

Synthesis was performed separately for the different tests.

Where possible, meta-analysis was conducted using the bivariate model with no covariates [[1](#_ENREF_1)].

Assessing the diagnostic performance of IHC is particularly challenging because it is (generally) a panel of four tests, and the results may be used to direct constitutional mutation testing.

The sensitivity of four-panel IHC was estimated according to the mutated gene, based on the genetic testing that would likely follow. Since *PMS2* and *MLH1* mutation testing are indicated by absent/abnormal PMS2 staining (even if MLH1 staining is normal), an absent/abnormal PMS2 stain was considered true positive for an *MLH1* mutation (likewise for MSH6 staining and *MSH2* mutations).

In considering test failure rate, we considered IHC to have failed if staining was unsuccessful for all proteins. If staining was successful for at least one protein, then the overall result was classed as true/false positive/negative based on the indicated testing.

## Results

### IHC

Eight studies [[2-9](#_ENREF_2)] were identified which were believed to provide estimates of the diagnostic performance of IHC at lower risk of bias.

The sensitivity and specificity of IHC were estimated to be 94.4% and 74.8% respectively (see Figure 1 and Table 1).


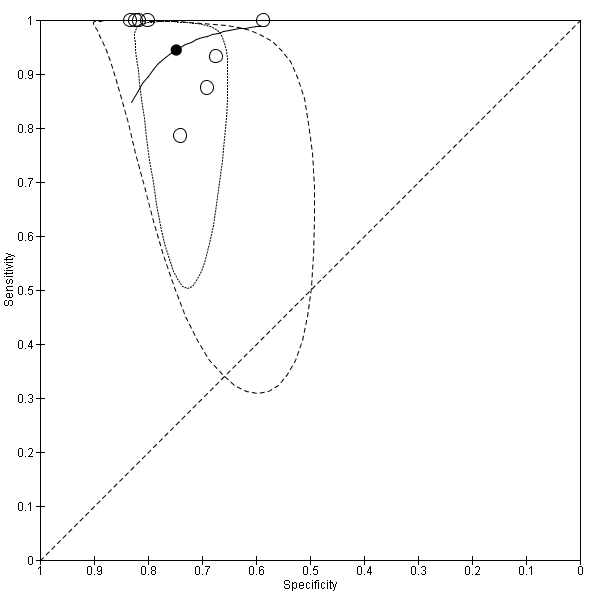


Figure 1: Meta-analysis of diagnostic performance of IHC

Table 1: Bivariate model results for IHC

| Parameter | Coefficient | Standard error |
| --- | --- | --- |
| Bivariate model |  |  |
| $\mathbb{E}\left[ \mathrm{logit} \left( Se \right) \right]$ | 2.832 | 0.879 |
| $\mathbb{E}\left[ \mathrm{logit} \left( Sp \right) \right]$ | 1.087 | 0.143 |
| $\mathrm{Var} \left[ \mathrm{logit} \left( Se \right) \right]$ | 0.516 | 1.473 |
| $\mathrm{Var} \left[ \mathrm{logit} \left( Sp \right) \right]$ | 0.102 | 0.082 |
| $\mathrm{Corr} \left[ \mathrm{logit} \left( Se \right),\mathrm{logit} \left( Sp \right) \right]$ | 0.948 | 0.819 |

The test failure rate was estimated from eight studies [[2-7](#_ENREF_2), [10](#_ENREF_10), [11](#_ENREF_11)] in which a total of 16 test failures were reported from 1 037 patients. A random effects logistic model was fitted to the data, with a mean estimate of 3.7%.

It was assumed that 89% of individuals with an *MLH1* mutation showing abnormalities on IHC would have absent/abnormal staining of MLH1 [[3-5](#_ENREF_3), [7-9](#_ENREF_7), [11-14](#_ENREF_11)], and 0.6% of individuals with non-*MLH1* mutations showing abnormalities on IHC would have absent/abnormal staining of MLH1 [[2-14](#_ENREF_2)]. For individuals without LS showing abnormalities on IHC it was assumed that 83% would have absent/abnormal staining of MLH1 [[2-14](#_ENREF_2)].

### MSI

Eight studies [[2-9](#_ENREF_2)] were identified which provided estimates of the diagnostic performance of MSI. Four of these [[2](#_ENREF_2), [7-9](#_ENREF_7)] categorised tumours as MSI if ≥1/5 markers showed instability, while the other four [[3-6](#_ENREF_3)] categorised tumours as MSI if ≥2/5 markers showed instability. No obvious threshold effect was observed, so all studies were pooled without including a covariate in the meta-analysis. One study [[9](#_ENREF_9)] showed very poor sensitivity and contributed to numerical issues in the meta-analysis, and it was excluded on this basis.

The sensitivity and specificity of MSI were estimated to be 90.3% and 77.1% respectively (see Figure 2 and Table 2).


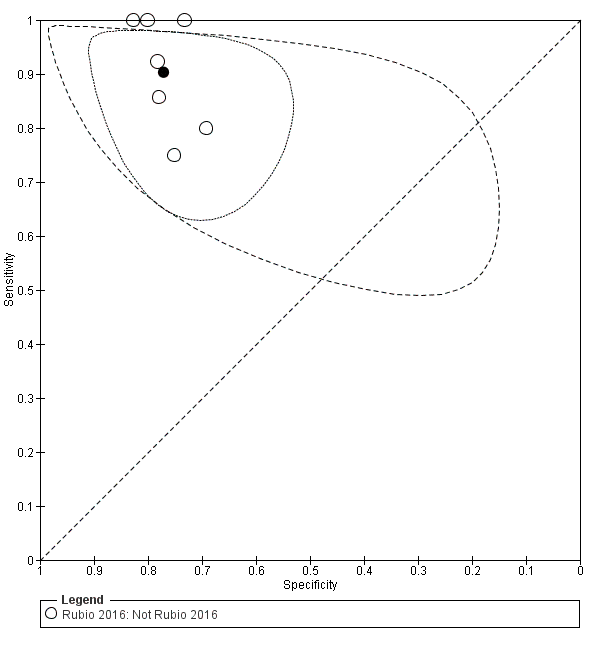


Figure 2: Meta-analysis of diagnostic performance of MSI

Table 2: Bivariate model results for MSI

| Parameter | Coefficient | Standard error |
| --- | --- | --- |
| Bivariate model |  |  |
| $\mathbb{E}\left[ \mathrm{logit} \left( Se \right) \right]$ | 2.233 | 0.501 |
| $\mathbb{E}\left[ \mathrm{logit} \left( Sp \right) \right]$ | 1.216 | 0.321 |
| $\mathrm{Var} \left[ \mathrm{logit} \left( Se \right) \right]$ | 0.197 | 0.000 |
| $\mathrm{Var} \left[ \mathrm{logit} \left( Sp \right) \right]$ | 0.652 | 0.000 |
| $\mathrm{Corr} \left[ \mathrm{logit} \left( Se \right),\mathrm{logit} \left( Sp \right) \right]$ | 1.000 | 0.000 |

The test failure rate was estimated from six studies [[3-8](#_ENREF_3)] in which a total of 12 failures were reported from 1 195 patients, with failure rates ranging from 0% to 5%. A random effects logistic model was fitted, with a mean estimate of 1.9%.

# References

1. Reitsma JB, Glas AS, Rutjes AW, Scholten RJ, Bossuyt PM, Zwinderman AH. Bivariate analysis of sensitivity and specificity produces informative summary measures in diagnostic reviews. J Clin Epidemiol. 2005;58(10):982-90. Epub 2005/09/20. doi: 10.1016/j.jclinepi.2005.02.022. PubMed PMID: 16168343.

2. Anagnostopoulos A, McKay VH, Cooper I, Campbell F, Greenhalgh L, Kirwan J. Identifying lynch syndrome in women presenting with endometrial carcinoma under the age of 50 years. Int J Gynecol Cancer. 2017;27(5):931-7. doi: 10.1097/IGC.0000000000000962.

3. Berends MJ, Wu Y, Sijmons RH, van der Sluis T, Ek WB, Ligtenberg MJ, et al. Toward new strategies to select young endometrial cancer patients for mismatch repair gene mutation analysis. J Clin Oncol. 2003;21(23):4364-70. Epub 2003/12/04. doi: 10.1200/JCO.2003.04.094. PubMed PMID: 14645426.

4. Egoavil C, Alenda C, Castillejo A, Paya A, Peiro G, Sánchez-Heras A-B, et al. Prevalence of Lynch Syndrome among Patients with Newly Diagnosed Endometrial Cancers. PLOS ONE. 2013;8(11):e79737. doi: 10.1371/journal.pone.0079737.

5. Ferguson SE, Aronson M, Pollett A, Eiriksson LR, Oza AM, Gallinger S, et al. Performance characteristics of screening strategies for Lynch syndrome in unselected women with newly diagnosed endometrial cancer who have undergone universal germline mutation testing. Cancer. 2014;120(24):3932-9. doi: 10.1002/cncr.28933.

6. Leenen CH, van Lier MG, van Doorn HC, van Leerdam ME, Kooi SG, de Waard J, et al. Prospective evaluation of molecular screening for Lynch syndrome in patients with endometrial cancer </= 70 years. Gynecol Oncol. 2012;125(2):414-20. Epub 2012/02/07. doi: 10.1016/j.ygyno.2012.01.049. PubMed PMID: 22306203.

7. Lu KH, Schorge JO, Rodabaugh KJ, Daniels MS, Sun CC, Soliman PT, et al. Prospective determination of prevalence of lynch syndrome in young women with endometrial cancer. J Clin Oncol. 2007;25(33):5158-64. Epub 2007/10/11. doi: 10.1200/JCO.2007.10.8597. PubMed PMID: 17925543.

8. Mercado RC, Hampel H, Kastrinos F, Steyerberg E, Balmana J, Stoffel E, et al. Performance of PREMM(1,2,6), MMRpredict, and MMRpro in detecting Lynch syndrome among endometrial cancer cases. Genet Med. 2012;14(7):670-80. Epub 2012/03/10. doi: 10.1038/gim.2012.18. PubMed PMID: 22402756; PubMed Central PMCID: PMC3396560.

9. Rubio I, Ibáñez-Feijoo E, Andrés L, Aguirre E, Balmaña J, Blay P, et al. Analysis of lynch syndrome mismatch repair genes in women with endometrial cancer. Oncology. 2016;91(3):171-6. doi: 10.1159/000447972.

10. Najdawi F, Crook A, Maidens J, McEvoy C, Fellowes A, Pickett J, et al. Lessons learnt from implementation of a Lynch syndrome screening program for patients with gynaecological malignancy. Pathology. 2017;49(5):457-64. doi: 10.1016/j.pathol.2017.05.004.

11. Watkins JC, Yang EJ, Muto MG, Feltmate CM, Berkowitz RS, Horowitz NS, et al. Universal screening for mismatch-repair deficiency in endometrial cancers to identify patients with lynch syndrome and lynch-like syndrome. Int J Gynecol Pathol. 2017;36(2):115-27. doi: 10.1097/PGP.0000000000000312.

12. Buchanan DD, Tan YY, Walsh MD, Clendenning M, Metcalf AM, Ferguson K, et al. Tumor mismatch repair immunohistochemistry and DNA MLH1 methylation testing of patients with endometrial cancer diagnosed at age younger than 60 years optimizes triage for population-level germline mismatch repair gene mutation testing. J Clin Oncol. 2014;32(2):90-100. Epub 2013/12/11. doi: 10.1200/JCO.2013.51.2129. PubMed PMID: 24323032; PubMed Central PMCID: PMC4876359.

13. Mas-Moya J, Dudley B, Brand RE, Thull D, Bahary N, Nikiforova MN, et al. Clinicopathological comparison of colorectal and endometrial carcinomas in patients with Lynch-like syndrome versus patients with Lynch syndrome. Hum Pathol. 2015;46(11):1616-25. doi: 10.1016/j.humpath.2015.06.022.

14. Moline J, Mahdi H, Yang B, Biscotti C, Roma AA, Heald B, et al. Implementation of tumor testing for lynch syndrome in endometrial cancers at a large academic medical center. Gynecol Oncol. 2013;130(1):121-6. doi: 10.1016/j.ygyno.2013.04.022.
