# Supplementary material for: Cost-effectiveness analysis of reflex testing for Lynch syndrome in women with endometrial cancer in the UK setting
Source: PLoS One. 2019 Aug 30;14(8):e0221419. doi: 10.1371/journal.pone.0221419 (PMC6716649; doi:10.1371/journal.pone.0221419)
Supplement: S5 Appendix — (DOCX) [file pone.0221419.s005.docx]

# S5 Appendix. Summary of model input parameters

Table 1 provides a summary of all model input parameters, including their base case value, PSA distribution (parameters and summary statistics) and sources. Table 2 and Table 3 additionally provide variance–covariance matrix for groups of parameters following a multivariate normal distribution.

Table 1: Summary of model input parameters

| Parameter | Base case value | PSA distribution | PSA Mean ± SD | Source |
| --- | --- | --- | --- | --- |
| Population |  |  |  |  |
| Number of relatives per proband | 6 | Γ(4, 1.5) | 6.0 ± 3.0 | [[1](#_ENREF_1)] |
| Proportion of relatives who are first-degree relatives of proband | 0.424 | Beta(269, 366) | 0.424 ± 0.020 | [[2-4](#_ENREF_2)] |
| Proportion of relatives receiving predictive testing found to have LS | 0.440 | Beta(371, 471) | 0.440 ± 0.017 | [[2-4](#_ENREF_2)] |
| Proportion of relatives who are women | 0.528 | Beta(312, 279) | 0.528 ± 0.021 | [[5](#_ENREF_5)] |
| Natural history |  |  |  |  |
| Prevalence of LS among all EC | 0.0391 | LogN(−3.33, 0.43) | 0.039 ± 0.017 | [[6-20](#_ENREF_6)] |
| Gene distribution among all EC (*MLH1* / *MSH2* / *MSH6* / *PMS2*) | 0.169  0.246  0.477  0.108 | Dirichlet(11, 16, 31, 7) | 0.169 ± 0.046  0.246 ± 0.053  0.477 ± 0.061  0.108 ± 0.038 | [[7](#_ENREF_7), [9-12](#_ENREF_9), [17](#_ENREF_17), [20-22](#_ENREF_20)] |
| CRC incidence with LS (lognormal distribution) |  |  |  | [[22-24](#_ENREF_22)] |
| …mu (baseline) | 4.306 | MVN (Table 2) | 4.306 ± 0.004 |  |
| …sigma | 0.567 |  | 0.567 ± 0.006 |  |
| …beta_MSH2 | 0.100 |  | 0.100 ± 0.005 |  |
| …beta_MSH6 | 0.531 |  | 0.531 ± 0.016 |  |
| …beta_PMS2 | 0.863 |  | 0.863 ± 0.110 |  |
| …beta_male | −0.118 |  | −0.118 ± 0.006 |  |
| …beta_prevcancer | −0.230 |  | −0.230 ± 0.010 |  |
| CRC incidence for women without LS  (table by age, per 100,000 person years) |  |  |  | [[25](#_ENREF_25)] |
| …Under 25 | 3.1 | LogN(−10.54, 0.57) | 3.1 ± 1.93 |  |
| …25–30 | 2.7 | LogN(−10.59, 0.39) | 2.7 ± 1.09 |  |
| …30–35 | 6.5 | LogN(−9.67, 0.23) | 6.5 ± 1.48 |  |
| …35–40 | 10.7 | LogN(−9.15, 0.16) | 10.7 ± 1.69 |  |
| …40–45 | 11.8 | LogN(−9.06, 0.15) | 11.8 ± 1.74 |  |
| …45–50 | 21.5 | LogN(−8.45, 0.10) | 21.5 ± 2.10 |  |
| …50–55 | 37.6 | LogN(−7.89, 0.07) | 37.6 ± 2.74 |  |
| …55–60 | 61.8 | LogN(−7.39, 0.06) | 61.8 ± 3.94 |  |
| …60–65 | 91.4 | LogN(−7.00, 0.05) | 91.4 ± 4.95 |  |
| …65–70 | 118.2 | LogN(−6.74, 0.04) | 118.2 ± 5.19 |  |
| …70–75 | 172.1 | LogN(−6.37, 0.04) | 172.1 ± 6.89 |  |
| …75–80 | 235.6 | LogN(−6.05, 0.04) | 235.6 ± 8.50 |  |
| …80–85 | 309.3 | LogN(−5.78, 0.03) | 309.3 ± 9.60 |  |
| …85–90 | 359.5 | LogN(−5.63, 0.03) | 359.5 ± 11.0 |  |
| …Over 90 | 304.2 | LogN(−5.80, 0.03) | 304.2 ± 10.3 |  |
| CRC incidence for men without LS  (table by age, per 100,000 person years) |  |  |  | [[25](#_ENREF_25)] |
| …Under 25 | 2.3 | LogN(−10.78, 0.45) | 2.3 ± 1.10 |  |
| …25–30 | 2.3 | LogN(−10.77, 0.42) | 2.3 ± 1.00 |  |
| …30–35 | 5.6 | LogN(−9.81, 0.20) | 5.6 ± 1.14 |  |
| …35–40 | 9.1 | LogN(−9.32, 0.16) | 9.1 ± 1.43 |  |
| …40–45 | 12.0 | LogN(−9.04, 0.12) | 12.0 ± 1.46 |  |
| …45–50 | 23.2 | LogN(−8.37, 0.10) | 23.2 ± 2.26 |  |
| …50–55 | 42.6 | LogN(−7.76, 0.07) | 42.6 ± 2.80 |  |
| …55–60 | 84.2 | LogN(−7.08, 0.05) | 84.2 ± 4.30 |  |
| …60–65 | 150.3 | LogN(−6.50, 0.04) | 150.3 ± 6.35 |  |
| …65–70 | 196.1 | LogN(−6.23, 0.03) | 196.1 ± 6.82 |  |
| …70–75 | 276.8 | LogN(−5.89, 0.03) | 276.8 ± 9.06 |  |
| …75–80 | 373.8 | LogN(−5.59, 0.03) | 373.8 ± 11.4 |  |
| …80–85 | 457.5 | LogN(−5.39, 0.03) | 457.5 ± 13.2 |  |
| …85–90 | 511.9 | LogN(−5.28, 0.03) | 511.9 ± 16.5 |  |
| …Over 90 | 460.3 | LogN(−5.38, 0.04) | 460.3 ± 19.4 |  |
| CRC mortality rate (without LS) |  |  |  | [[26](#_ENREF_26)] |
| …Stage I | 0.014 | LogN(−4.26, 0.055) | 0.014 ± 0.001 |  |
| …Stage II | 0.052 | LogN(−2.95, 0.014) | 0.052 ± 0.001 |  |
| …Stage III | 0.148 | LogN(−1.91, 0.009) | 0.148 ± 0.001 |  |
| …Stage IV | 0.544 | LogN(−0.61, 0.013) | 0.544 ± 0.007 |  |
| CRC mortality hazard ratio with LS (Stages I–III) | 0.660 | LogN(−0.479, 0.357) | 0.660 ± 0.244 | [[27](#_ENREF_27)] |
| EC mortality rate with LS | 0.004 | LogN(−6.07, 1.06) | 0.004 ± 0.006 | [[22](#_ENREF_22)] |
| EC mortality rate without LS (by age) |  |  |  | [[28](#_ENREF_28)] |
| ...15–45 | 0.026 | LogN(−3.63, 0.100) | 0.027 ± 0.003 |  |
| …45–55 | 0.028 | LogN(−3.57, 0.055) | 0.028 ± 0.002 |  |
| …55–65 | 0.031 | LogN(−3.46, 0.036) | 0.031 ± 0.001 |  |
| …65–75 | 0.048 | LogN(−3.03, 0.031) | 0.048 ± 0.002 |  |
| …Over 75 | 0.092 | LogN(−2.38, 0.034) | 0.092 ± 0.003 |  |
| Effectiveness of risk reduction |  |  |  |  |
| Age range for surveillance colonoscopy | 25–75 |  |  |  |
| Interval between colonoscopies | 2.1 | LogN(0.726, 0.180) | 2.1 ± 0.382 |  |
| Uptake of colonoscopy if diagnosed LS | 0.972 | Beta(105, 3) | 0.972 ± 0.016 | [[5](#_ENREF_5)] |
| Uptake of colonoscopy if diagnosed PLS | 0.701 | Beta(68, 29) | 0.701 ± 0.046 | [[5](#_ENREF_5)] |
| Hazard ratio for CRC incidence if undergoing colonoscopy | 0.387 | LogN(−1.031, 0.405) | 0.387 ± 0.163 | [[4](#_ENREF_4), [29](#_ENREF_29)] |
| CRC stage distribution in surveillance |  | Dirichlet |  | [[30](#_ENREF_30)] |
| …Stage I | 0.686 | 29.5 | 0.686 ± 0.070 |  |
| …Stage II | 0.105 | 4.5 | 0.105 ± 0.046 |  |
| …Stage III | 0.128 | 5.5 | 0.128 ± 0.050 |  |
| …Stage IV | 0.081 | 3.5 | 0.081 ± 0.041 |  |
| CRC stage distribution not in surveillance (sporadic) |  | Dirichlet |  |  |
| …Stage I | 0.176 | 5989.5 | 0.176 ± 0.002 |  |
| …Stage II | 0.270 | 9170.5 | 0.270 ± 0.002 |  |
| …Stage III | 0.295 | 10036.5 | 0.295 ± 0.002 |  |
| …Stage IV | 0.259 | 8816.5 | 0.259 ± 0.002 |  |
| CRC stage distribution not in surveillance (LS) |  | Dirichlet |  | [[31](#_ENREF_31)] |
| …Stage I | 0.188 | 7.5 | 0.188 ± 0.061 |  |
| …Stage II | 0.488 | 19.5 | 0.488 ± 0.078 |  |
| …Stage III | 0.213 | 8.5 | 0.213 ± 0.064 |  |
| …Stage IV | 0.113 | 4.5 | 0.113 ± 0.049 |  |
| Diagnostic accuracy |  |  |  |  |
| Sensitivity of IHC | 0.944 | Bivariate logit normal (Table 3) | 0.944 ± 0.060 | [[6](#_ENREF_6), [8](#_ENREF_8), [10-13](#_ENREF_10), [15](#_ENREF_15), [19](#_ENREF_19)] |
| Specificity of IHC | 0.748 |  | 0.748 ± 0.066 | [[6](#_ENREF_6), [8](#_ENREF_8), [10-13](#_ENREF_10), [15](#_ENREF_15), [19](#_ENREF_19)] |
| Probability of abnormal MLH1 given dMMR and pathogenic *MLH1* mutation | 0.889 | Beta(16, 2) | 0.889 ± 0.072 | [[8-11](#_ENREF_8), [13-15](#_ENREF_13), [19](#_ENREF_19), [20](#_ENREF_20), [32](#_ENREF_32)] |
| Probability of abnormal MLH1 given dMMR and other LS mutation | 0.006 | Beta(0.5, 79.5) | 0.006 ± 0.009 | [[6](#_ENREF_6), [8-15](#_ENREF_8), [17](#_ENREF_17), [19](#_ENREF_19), [20](#_ENREF_20), [32](#_ENREF_32)] |
| Probability of abnormal MLH1 given dMMR and sporadic | 0.828 | Beta(371, 77) | 0.828 ± 0.018 | [[6](#_ENREF_6), [8-15](#_ENREF_8), [17](#_ENREF_17), [19](#_ENREF_19), [20](#_ENREF_20), [32](#_ENREF_32)] |
| IHC test failure rate | 0.009 | Beta(0.282, 29.77) | 0.009 ± 0.017 | [[6](#_ENREF_6), [8](#_ENREF_8), [10-13](#_ENREF_10), [17](#_ENREF_17), [20](#_ENREF_20)] |
| Sensitivity of MSI | 0.903 | Bivariate logit normal (Table 3) | 0.903 ± 0.059 | [[6](#_ENREF_6), [8](#_ENREF_8), [10-13](#_ENREF_10), [15](#_ENREF_15)] |
| Specificity of MSI | 0.771 |  | 0.771 ± 0.153 | [[6](#_ENREF_6), [8](#_ENREF_8), [10-13](#_ENREF_10), [15](#_ENREF_15)] |
| MSI test failure rate | 0.009 | Beta(0.605, 68.41) | 0.009 ± 0.011 | [[8](#_ENREF_8), [10-13](#_ENREF_10), [15](#_ENREF_15)] |
| Probability of *MLH1* methylation given MLH1 abnormal and |  |  |  |  |
| …Pathogenic *MLH1* mutation | 0.050 | Beta(1, 19) | 0.050 ± 0.048 | Assumed |
| ...Other LS mutation | 0.936 | Beta(39.75, 2.73) | 0.936 ± 0.037 | Assumed |
| …Sporadic | 0.936 | Beta(39.75, 2.73) | 0.936 ± 0.037 | [[6](#_ENREF_6), [9](#_ENREF_9), [10](#_ENREF_10), [12](#_ENREF_12), [13](#_ENREF_13), [17](#_ENREF_17), [20](#_ENREF_20)] |
| Probability of *MLH1* methylation given MSI and |  |  |  |  |
| …Pathogenic *MLH1* mutation | 0.050 | Beta(1, 19) | 0.050 ± 0.048 | Assumed |
| ...Other LS mutation | 0.071 | Beta(1, 13) | 0.071 ± 0.066 | [[9](#_ENREF_9)] |
| …Sporadic | 0.670 | LogN(−0.40, 0.055) | 0.670 ± 0.037 | [[9](#_ENREF_9)] |
| Family history (HSROC model) |  |  |  | Assumed |
| …theta (positivity parameter) | −1 | N(−1, 2) | −1 ± 2 |  |
| …alpha (discriminative ability) | 0.223 | LogN(−2, 1) | 0.223 ± 0.292 |  |
| …beta (asymmetry) | 0 | N(0, 1) | 0 ± 1 |  |
| …(Derived sensitivity) | 0.291 |  |  |  |
| …(Derived specificity) | 0.752 |  |  |  |
| Diagnostic MMR mutation testing |  |  |  |  |
| …Acceptance of counselling (tumour-testing strategies) | 0.554 | Beta(2.66, 2.14) | 0.554 ± 0.206 | [[33](#_ENREF_33)] |
| …Acceptance of counselling (direct testing) | 0.5 | Beta(4, 4) | 0.500 ± 0.167 | Assumed |
| …Acceptance of diagnostic testing (given accepted counselling) | 0.9 | Beta(9, 1) | 0.900 ± 0.090 | Assumed |
| …Sensitivity | 0.9 | Beta(9, 1) | 0.900 ± 0.090 | Assumed |
| …Specificity | 1 | N/A | N/A | Assumed |
| Predictive MMR mutation testing |  |  |  |  |
| …Acceptance of counselling | 0.777 | $X + Y\left( 1-X \right)$  *X* ~ Beta(329, 262)  *Y* ~ Beta(97, 98) | 0.778 ± 0.019 | [[5](#_ENREF_5)] |
| …Acceptance of predictive testing (given accepted counselling) | 0.716 | $X/\left( X+Y\left( 1-X \right) \right)$  *X*, *Y* as above | 0.716 ± 0.022 | [[5](#_ENREF_5)] |
| Costs |  |  |  |  |
| IHC | 210 | Γ(25, 8.4) | 210 ± 42 | [[1](#_ENREF_1)] |
| MSI | 202 | Γ(25, 8.08) | 202 ± 40.4 | [[1](#_ENREF_1)] |
| Methylation | 136 | Γ(25, 5.44) | 136 ± 27.2 | [[1](#_ENREF_1)] |
| Offer counselling | 27.3 | LogN(3.27, 0.280) | 27.3 ± 7.80 |  |
| Pre-test counselling (proband) | 347 | Γ(25, 13.9) | 347 ± 69.5 | [[34](#_ENREF_34)] |
| Diagnostic MMR mutation testing | 755 | Γ(25, 30.2) | 755 ± 151 | [[35](#_ENREF_35)] |
| Post-test counselling (proband) | 133 | Γ(25, 5.33) | 133 ± 26.6 | [[34](#_ENREF_34)] |
| GP appointment | 36.4 | Γ(25, 1.46) | 36.4 ± 7.28 | [[36](#_ENREF_36)] |
| Pre-test counselling (relative) | 172 | Γ(25, 6.87) | 172 ± 34.4 | [[34](#_ENREF_34)] |
| Predictive MMR mutation testing | 166 | Γ(25, 6.65) | 166 ± 33.3 | [[35](#_ENREF_35)] |
| Post-test counselling (relative) | 133 | Γ(25, 5.33) | 133 ± 26.6 | [[34](#_ENREF_34)] |
| Colonoscopy | 583 | Γ(25, 23.3) | 583 ± 117 | [[37](#_ENREF_37)] |
| Stage I CRC (by age) |  |  |  | [[38](#_ENREF_38)] |
| …40–49 | 8754 | Γ(25, 350) | 8754 ± 1751 |  |
| …50–59 | 5712 | Γ(25, 228) | 5712 ± 1142 |  |
| …60–69 | 4623 | Γ(25, 185) | 4623 ± 925 |  |
| …70–79 | 3178 | Γ(25, 127) | 3178 ± 636 |  |
| …80–100 | 1380 | Γ(25, 55.2) | 1380 ± 276 |  |
| Stage II CRC (by age) |  |  |  |  |
| …40–49 | 8741 | Γ(25, 350) | 8741 ± 1748 |  |
| …50–59 | 7016 | Γ(25, 281) | 7016 ± 1403 |  |
| …60–69 | 5352 | Γ(25, 214) | 5352 ± 1070 |  |
| …70–79 | 3455 | Γ(25, 138) | 3455 ± 691 |  |
| …80–100 | 1546 | Γ(25, 61.8) | 1546 ± 309 |  |
| Stage III CRC (by age) |  |  |  |  |
| …40–49 | 14490 | Γ(25, 580) | 14490 ± 2898 |  |
| …50–59 | 9692 | Γ(25, 388) | 9692 ± 1938 |  |
| …60–69 | 7259 | Γ(25, 290) | 7259 ± 1452 |  |
| …70–79 | 4485 | Γ(25, 179) | 4485 ± 897 |  |
| …80–100 | 1561 | Γ(25, 62.4) | 1561 ± 312 |  |
| Stage IV CRC (by age) |  |  |  |  |
| …40–49 | 11705 | Γ(25, 468) | 11705 ± 2341 |  |
| …50–59 | 8444 | Γ(25, 338) | 8444 ± 1689 |  |
| …60–69 | 6509 | Γ(25, 260) | 6509 ± 1302 |  |
| …70–79 | 4365 | Γ(25, 175) | 4365 ± 873 |  |
| …80–100 | 807 | Γ(25, 32.3) | 807 ± 161 |  |
| Utilities |  |  |  |  |
| Baseline utility model |  |  |  | [[39](#_ENREF_39)] |
| …Intercept | 0.9509 | N(0.9509, 0.0089) | 0.9509 ± 0.0089 |  |
| …Male | 0.0212 | N(0.0212, 0.0027) | 0.0212 ± 0.0027 |  |
| …Age | −0.0003 | N(−0.0003, 0.0004) | −0.0003 ± 0.0004 |  |
| …Age² | −3.32 × 10^−5^ | N(−3.32×10^−5^, 1.69×10^−5^) | (−3.32 ± 1.69) × 10^−5^ |  |
| …(Resulting baseline utility for proband at start) | 0.816 |  |  |  |
| …(Resulting baseline utility for relative at start) | 0.850 |  |  |  |
| Impact of testing on HRQoL (multipliers) |  |  |  | [[40](#_ENREF_40)] |
| …Declining counselling | 1 | N(1, 0.05) | 1 ± 0.05 | Assumed |
| …Declining genetic testing | 1 | N(1, 0.05) | 1 ± 0.05 | Assumed |
| …Diagnosed with LS | 1 | N(1, 0.05) | 1 ± 0.05 | Assumed |
| …Diagnosed with putative LS | 1 | N(1, 0.05) | 1 ± 0.05 | Assumed |
| Colorectal cancer (multipliers) |  |  |  |  |
| …Stage I | 1 |  |  | Assumed |
| …Stage II | 1 |  |  | Assumed |
| …Stage III | 1 |  |  | Assumed |
| …Stage IV | 0.789 | Beta(134, 36.0) | 0.789 ± 0.031 | [[41](#_ENREF_41)] |
| Endometrial cancer (multiplier) | 1 |  |  | Assumed |

Table 2: Variance–covariance matrix for colorectal cancer incidence model

| Parameter | MSH2 | MSH6 | PMS2 | M0 | U1 | (Intercept) | ln_sigma |
| --- | --- | --- | --- | --- | --- | --- | --- |
| MSH2 | 0.004861 |  |  |  |  |  |  |
| MSH6 | 0.002427 | 0.016159 |  |  |  |  |  |
| PMS2 | 0.003063 | 0.006487 | 0.110071 |  |  |  |  |
| M0 | -2.8E-05 | -0.00039 | -0.0008 | 0.005788 |  |  |  |
| U1 | -0.00137 | -0.00359 | -0.00651 | 0.003565 | 0.009597 |  |  |
| (Intercept) | -0.00129 | -0.00076 | -2.4E-05 | -0.00306 | -0.00361 | 0.00364 |  |
| ln_sigma | 0.00147 | 0.005272 | 0.009192 | -0.00132 | -0.00627 | 0.001641 | 0.010197 |

Table 3: Variance–covariance matrices for diagnostic performance of IHC and MSI

| Parameter | Logit(sensitivity) | Logit(specificity) |
| --- | --- | --- |
| IHC | | |
| Logit(sensitivity) | 1.076805 |  |
| Logit(specificity) | 0.258114 | 0.126149 |
| MSI | | |
| Logit(sensitivity) | 0.432789 |  |
| Logit(specificity) | 0.459689 | 0.921361 |

# References

1. Snowsill T, Coelho H, Huxley N, Jones-Hughes T, Briscoe S, Frayling IM, et al. Molecular testing for Lynch syndrome in people with colorectal cancer: systematic reviews and economic evaluation. Health Technol Assess. 2017;21(51):1-238. Epub 2017/09/13. doi: 10.3310/hta21510. PubMed PMID: 28895526; PubMed Central PMCID: PMC5611555.

2. Hampel H, Frankel WL, Martin E, Arnold M, Khanduja K, Kuebler P, et al. Feasibility of screening for Lynch syndrome among patients with colorectal cancer. J Clin Oncol. 2008;26(35):5783-8. Epub 2008/09/24. doi: 10.1200/JCO.2008.17.5950. PubMed PMID: 18809606; PubMed Central PMCID: PMC2645108.

3. Jenkins MA, Baglietto L, Dowty JG, Van Vliet CM, Smith L, Mead LJ, et al. Cancer risks for mismatch repair gene mutation carriers: a population-based early onset case-family study. Clin Gastroenterol Hepatol. 2006;4(4):489-98. Epub 2006/04/18. doi: 10.1016/j.cgh.2006.01.002. PubMed PMID: 16616355.

4. Snowsill T, Huxley N, Hoyle M, Jones-Hughes T, Coelho H, Cooper C, et al. A systematic review and economic evaluation of diagnostic strategies for Lynch syndrome. Health Technol Assess. 2014;18(58):1-406. Epub 2014/09/23. doi: 10.3310/hta18580. PubMed PMID: 25244061; PubMed Central PMCID: PMC4781313.

5. Barrow P. Hereditary colorectal cancer: registration, screening and prognostic biomarker analysis. Manchester: University of Manchester; 2015.

6. Anagnostopoulos A, McKay VH, Cooper I, Campbell F, Greenhalgh L, Kirwan J. Identifying lynch syndrome in women presenting with endometrial carcinoma under the age of 50 years. Int J Gynecol Cancer. 2017;27(5):931-7. doi: 10.1097/IGC.0000000000000962.

7. Batte BAL, Bruegl AS, Daniels MS, Ring KL, Dempsey KM, Djordjevic B, et al. Consequences of universal MSI/IHC in screening endometrial cancer patients for Lynch syndrome. Gynecol Oncol. 2014;134(2):319-25. doi: 10.1016/j.ygyno.2014.06.009.

8. Berends MJ, Wu Y, Sijmons RH, van der Sluis T, Ek WB, Ligtenberg MJ, et al. Toward new strategies to select young endometrial cancer patients for mismatch repair gene mutation analysis. J Clin Oncol. 2003;21(23):4364-70. Epub 2003/12/04. doi: 10.1200/JCO.2003.04.094. PubMed PMID: 14645426.

9. Buchanan DD, Tan YY, Walsh MD, Clendenning M, Metcalf AM, Ferguson K, et al. Tumor mismatch repair immunohistochemistry and DNA MLH1 methylation testing of patients with endometrial cancer diagnosed at age younger than 60 years optimizes triage for population-level germline mismatch repair gene mutation testing. J Clin Oncol. 2014;32(2):90-100. Epub 2013/12/11. doi: 10.1200/JCO.2013.51.2129. PubMed PMID: 24323032; PubMed Central PMCID: PMC4876359.

10. Egoavil C, Alenda C, Castillejo A, Paya A, Peiro G, Sánchez-Heras A-B, et al. Prevalence of Lynch Syndrome among Patients with Newly Diagnosed Endometrial Cancers. PLOS ONE. 2013;8(11):e79737. doi: 10.1371/journal.pone.0079737.

11. Ferguson SE, Aronson M, Pollett A, Eiriksson LR, Oza AM, Gallinger S, et al. Performance characteristics of screening strategies for Lynch syndrome in unselected women with newly diagnosed endometrial cancer who have undergone universal germline mutation testing. Cancer. 2014;120(24):3932-9. doi: 10.1002/cncr.28933.

12. Leenen CH, van Lier MG, van Doorn HC, van Leerdam ME, Kooi SG, de Waard J, et al. Prospective evaluation of molecular screening for Lynch syndrome in patients with endometrial cancer </= 70 years. Gynecol Oncol. 2012;125(2):414-20. Epub 2012/02/07. doi: 10.1016/j.ygyno.2012.01.049. PubMed PMID: 22306203.

13. Lu KH, Schorge JO, Rodabaugh KJ, Daniels MS, Sun CC, Soliman PT, et al. Prospective determination of prevalence of lynch syndrome in young women with endometrial cancer. J Clin Oncol. 2007;25(33):5158-64. Epub 2007/10/11. doi: 10.1200/JCO.2007.10.8597. PubMed PMID: 17925543.

14. Mas-Moya J, Dudley B, Brand RE, Thull D, Bahary N, Nikiforova MN, et al. Clinicopathological comparison of colorectal and endometrial carcinomas in patients with Lynch-like syndrome versus patients with Lynch syndrome. Hum Pathol. 2015;46(11):1616-25. doi: 10.1016/j.humpath.2015.06.022.

15. Mercado RC, Hampel H, Kastrinos F, Steyerberg E, Balmana J, Stoffel E, et al. Performance of PREMM(1,2,6), MMRpredict, and MMRpro in detecting Lynch syndrome among endometrial cancer cases. Genet Med. 2012;14(7):670-80. Epub 2012/03/10. doi: 10.1038/gim.2012.18. PubMed PMID: 22402756; PubMed Central PMCID: PMC3396560.

16. Mills AM, Liou S, Ford JM, Berek JS, Pai RK, Longacre TA. Lynch syndrome screening should be considered for all patients with newly diagnosed endometrial cancer. Am J Surg Pathol. 2014;38(11):1501-9. doi: 10.1097/PAS.0000000000000321.

17. Najdawi F, Crook A, Maidens J, McEvoy C, Fellowes A, Pickett J, et al. Lessons learnt from implementation of a Lynch syndrome screening program for patients with gynaecological malignancy. Pathology. 2017;49(5):457-64. doi: 10.1016/j.pathol.2017.05.004.

18. Ring KL, Bruegl AS, Allen BA, Elkin EP, Singh N, Hartman AR, et al. Germline multi-gene hereditary cancer panel testing in an unselected endometrial cancer cohort. Mod Pathol. 2016;29(11):1381-9. doi: 10.1038/modpathol.2016.135.

19. Rubio I, Ibáñez-Feijoo E, Andrés L, Aguirre E, Balmaña J, Blay P, et al. Analysis of lynch syndrome mismatch repair genes in women with endometrial cancer. Oncology. 2016;91(3):171-6. doi: 10.1159/000447972.

20. Watkins JC, Yang EJ, Muto MG, Feltmate CM, Berkowitz RS, Horowitz NS, et al. Universal screening for mismatch-repair deficiency in endometrial cancers to identify patients with lynch syndrome and lynch-like syndrome. Int J Gynecol Pathol. 2017;36(2):115-27. doi: 10.1097/PGP.0000000000000312.

21. Hampel H, Frankel W, Panescu J, Lockman J, Sotamaa K, Fix D, et al. Screening for Lynch syndrome (hereditary nonpolyposis colorectal cancer) among endometrial cancer patients. Cancer Res. 2006;66(15):7810-7. Epub 2006/08/04. doi: 10.1158/0008-5472.CAN-06-1114. PubMed PMID: 16885385.

22. Moller P, Seppala T, Bernstein I, Holinski-Feder E, Sala P, Evans DG, et al. Cancer incidence and survival in Lynch syndrome patients receiving colonoscopic and gynaecological surveillance: first report from the prospective Lynch syndrome database. Gut. 2017;66(3):464-72. Epub 2015/12/15. doi: 10.1136/gutjnl-2015-309675. PubMed PMID: 26657901; PubMed Central PMCID: PMC5534760.

23. Moller P, Seppala T, Bernstein I, Holinski-Feder E, Sala P, Evans DG, et al. Incidence of and survival after subsequent cancers in carriers of pathogenic MMR variants with previous cancer: a report from the prospective Lynch syndrome database. Gut. 2017;66(9):1657-64. Epub 2016/06/05. doi: 10.1136/gutjnl-2016-311403. PubMed PMID: 27261338; PubMed Central PMCID: PMC5561364.

24. Moller P, Seppala TT, Bernstein I, Holinski-Feder E, Sala P, Evans DG, et al. Cancer risk and survival in path_MMR carriers by gene and gender up to 75 years of age: a report from the Prospective Lynch Syndrome Database. Gut. 2017. Epub 2017/07/30. doi: 10.1136/gutjnl-2017-314057. PubMed PMID: 28754778.

25. Office for National Statistics. Cancer registration statistics, England: 2015 2017 [cited 2017 December 15]. Available from: <https://www.ons.gov.uk/peoplepopulationandcommunity/healthandsocialcare/conditionsanddiseases/datasets/cancerregistrationstatisticscancerregistrationstatisticsengland>.

26. National Cancer Intelligence Network. Colorectal cancer survival by stage 2009 [cited 2017 September 4]. Available from: <http://www.ncin.org.uk/publications/data_briefings/colorectal_cancer_survival_by_stage>.

27. Lautrup CK, Mikkelsen EM, Lash TL, Katballe N, Sunde L. Survival in familial colorectal cancer: a Danish cohort study. Familial Cancer. 2015;14(4):553-9. doi: 10.1007/s10689-015-9812-1.

28. Office for National Statistics, Public Health England. Cancer Survival in England: adults diagnosed between 2011 and 2015 and followed up to 2016 2017 [cited 2017 December 18]. Available from: <https://www.ons.gov.uk/peoplepopulationandcommunity/healthandsocialcare/conditionsanddiseases/datasets/cancersurvivalratescancersurvivalinenglandadultsdiagnosed>.

29. Jarvinen HJ, Aarnio M, Mustonen H, Aktan-Collan K, Aaltonen LA, Peltomaki P, et al. Controlled 15-year trial on screening for colorectal cancer in families with hereditary nonpolyposis colorectal cancer. Gastroenterology. 2000;118(5):829-34. Epub 2000/04/28. PubMed PMID: 10784581.

30. Mecklin JP, Aarnio M, Laara E, Kairaluoma MV, Pylvanainen K, Peltomaki P, et al. Development of colorectal tumors in colonoscopic surveillance in Lynch syndrome. Gastroenterology. 2007;133(4):1093-8. Epub 2007/10/09. doi: 10.1053/j.gastro.2007.08.019. PubMed PMID: 17919485.

31. Barnetson RA, Tenesa A, Farrington SM, Nicholl ID, Cetnarskyj R, Porteous ME, et al. Identification and survival of carriers of mutations in DNA mismatch-repair genes in colon cancer. N Engl J Med. 2006;354(26):2751-63. Epub 2006/06/30. doi: 10.1056/NEJMoa053493. PubMed PMID: 16807412.

32. Moline J, Mahdi H, Yang B, Biscotti C, Roma AA, Heald B, et al. Implementation of tumor testing for lynch syndrome in endometrial cancers at a large academic medical center. Gynecol Oncol. 2013;130(1):121-6. doi: 10.1016/j.ygyno.2013.04.022.

33. Heald B, Plesec T, Liu X, Pai R, Patil D, Moline J, et al. Implementation of universal microsatellite instability and immunohistochemistry screening for diagnosing lynch syndrome in a large academic medical center. J Clin Oncol. 2013;31(10):1336-40. Epub 2013/02/13. doi: 10.1200/JCO.2012.45.1674. PubMed PMID: 23401454; PubMed Central PMCID: PMC4878100.

34. Slade I, Hanson H, George A, Kohut K, Strydom A, Wordsworth S, et al. A cost analysis of a cancer genetic service model in the UK. Journal of Community Genetics. 2016;7(3):185-94. doi: 10.1007/s12687-016-0266-4.

35. UK Genetic Testing Network. Colorectal cancer, hereditary nonpolyposis and Lynch syndrome 2018 [cited 2018 February 14]. Available from: <https://ukgtn.nhs.uk/find-a-test/search-by-disorder-gene/colorectal-cancer-hereditary-nonpolyposis-and-lynch-syndrome-534/>.

36. Curtis L, Burns A. Unit Costs of Health and Social Care 2016. Canterbury: Personal Social Services Research Unit, University of Kent; 2016.

37. Department of Health. NHS reference costs 2015 to 2016. 2016 [cited 2017 December 6]. Available from: <https://www.gov.uk/government/publications/nhs-reference-costs-2015-to-2016>.

38. Whyte S, Harnan S, Scope A, Simpson E, Tappenden P, Duffy S, et al. Early awareness interventions for cancer: Colorectal cancer. University of Sheffield and University of York: Economic Evaluation of Health and Social Care Interventions Policy Research Unit, 2012.

39. Ara R, Brazier JE. Populating an Economic Model with Health State Utility Values: Moving toward Better Practice. Value in Health. 2010;13(5):509-18. doi: 10.1111/j.1524-4733.2010.00700.x.

40. Kuppermann M, Wang G, Wong S, Blanco A, Conrad P, Nakagawa S, et al. Preferences for outcomes associated with decisions to undergo or forgo genetic testing for Lynch syndrome. Cancer. 2013;119(1):215-25. Epub 2012/07/13. doi: 10.1002/cncr.27634. PubMed PMID: 22786716; PubMed Central PMCID: PMC4356667.

41. Djalalov S, Rabeneck L, Tomlinson G, Bremner KE, Hilsden R, Hoch JS. A Review and Meta-analysis of Colorectal Cancer Utilities. Med Decis Making. 2014;34(6):809-18. Epub 2014/06/07. doi: 10.1177/0272989X14536779. PubMed PMID: 24903121.
