# Supplementary material for: Cost-effectiveness analysis of reflex testing for Lynch syndrome in women with endometrial cancer in the UK setting
Source: PLoS One. 2019 Aug 30;14(8):e0221419. doi: 10.1371/journal.pone.0221419 (PMC6716649; doi:10.1371/journal.pone.0221419)
Supplement: S6 Appendix — (DOCX) [file pone.0221419.s006.docx]

# S6 Appendix. Scenario analyses

## Summary

Six scenario analyses were conducted to investigate the potential impact of parameter and structural uncertainty. As shown in Table 1, IHC with *MLH1* methylation testing remained cost-effective for all but one of these scenarios.

Table 1: Summary of results of scenario analyses

| Scenario | ICER (IHC with *MLH1* methylation testing vs. No testing) [£/QALY] | Cost-effective strategy (at £20 000 per QALY threshold) |
| --- | --- | --- |
| Base case | 14 200 | IHC with *MLH1* methylation |
| Prevalence estimates from low drop-out studies | 16 800 | IHC with *MLH1* methylation |
| Weibull model for CRC incidence | 13 800 | IHC with *MLH1* methylation |
| Delayed CRC surveillance for *MSH6* and *PMS2* | 14 300 | IHC with *MLH1* methylation |
| CRC incidence reduction due to colonoscopic surveillance estimated from Arrigoni et al. 2005 [[1](#_ENREF_1)] | 52 500 | No testing |
| Likelihood of *MLH1* methylation following MSI-H from Hampel et al. 2006 [[2](#_ENREF_2)] | 14 200 | IHC with *MLH1* methylation |
| Acceptance of genetic counselling following tumour-based testing estimated from Batte et al. 2014 [[3](#_ENREF_3)] | 16 700 | IHC with *MLH1* methylation |
| Costs of CRC based on Murphy and Gray 2015 [[4](#_ENREF_4)] | 11 700 | IHC with *MLH1* methylation |
| Health state utility values related to genetic counselling and testing based on Kuppermann et al. 2013 [[5](#_ENREF_5)] | 15 100 | IHC with *MLH1* methylation |
| Health state utility values for CRC based on Ness et al. 1999 [[6](#_ENREF_6)] | 13 700 | IHC with *MLH1* methylation |
| Risk prediction tools incorporated based on Mercado et al. 2012 [[7](#_ENREF_7)] | 14 200 | IHC with *MLH1* methylation |
| Cost of diagnostic mutation testing £525 following IHC | 13 900 | IHC with *MLH1* methylation |

## Prevalence estimates from low drop-out studies

In this scenario, the overall prevalence of Lynch syndrome among women with endometrial cancer (of all ages) was estimated from studies where fewer than 10% of participants with suggestive tumour-based tests were not tested for constitutional MMR mutations. This was the case in eight of the 15 studies. The resulting prevalence estimate was 3.0% (compared to 3.9% when estimated from all 15 studies). As would be expected, when prevalence is lower, the cost-effectiveness of testing is worsened, since more individuals need to be tested to identify each case of Lynch syndrome, although the ICER for IHC with methylation remains below £20 000 per QALY (see Table 2).

Table 2: Prevalence estimates from low drop-out studies - cost-effectiveness results

| Strategy | Incremental QALYs vs. no testing | Incremental costs vs. no testing (£) | ICER vs. no testing (£/QALY) | Fully incremental ICER (£/QALY) |
| --- | --- | --- | --- | --- |
| MSI with methylation | 26.5 | 501 800 | 18 900 | Dominated |
| Direct mutation testing | 26.8 | 729 000 | 27 200 | Dominated |
| IHC with methylation | 29.1 | 490 000 | 16 800 | 16 800 |
| MSI | 29.7 | 728 000 | 24 500 | Extendedly Dominated |
| IHC | 31.2 | 781 000 | 25 000 | 136 000 |

## Weibull model for CRC incidence

A Weibull model was fitted to CRC incidence data from the Prospective Lynch Syndrome Database [[8-10](#_ENREF_8)], as described in **S3 Appendix**. The AIC for the Weibull model was third lowest, with the log-logistic model performing better; however, the log-logistic and log-normal models frequently produce very similar curves, while the Weibull hazard function is qualitatively very different (see Figure 1), while retaining reasonable predictive performance.


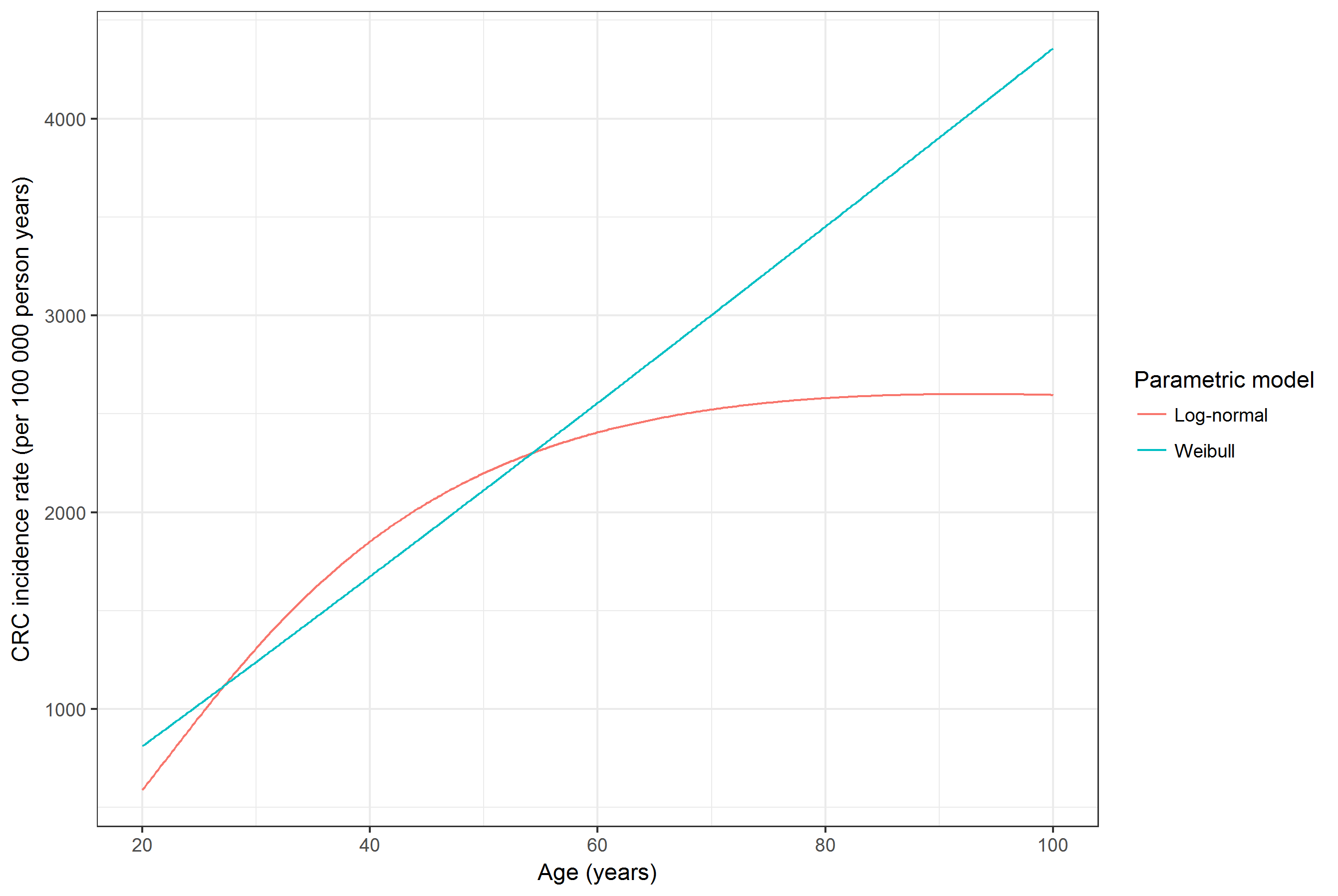


Figure 1: Comparison of log-normal (base case) and Weibull (scenario analysis) CRC incidence models

Note: Figure shows modelled incidence rate for male MLH1 mutation carriers with previous cancer (the highest risk group in both parametric models)

The use of the Weibull model has a very limited impact on cost-effectiveness results (see Table 3), suggesting that the results are robust to different statistical modelling of this key model input.

Table 3: Weibull model for CRC incidence - cost-effectiveness results

| Strategy | Incremental QALYs vs. no testing | Incremental costs vs. no testing (£) | ICER vs. no testing (£/QALY) | Fully incremental ICER (£/QALY) |
| --- | --- | --- | --- | --- |
| MSI with methylation | 35.3 | 544 000 | 15 400 | Dominated |
| Direct mutation testing | 35.9 | 768 000 | 21 400 | Dominated |
| IHC with methylation | 38.8 | 537 000 | 13 800 | 13 800 |
| MSI | 39.2 | 770 000 | 19 700 | Extendedly dominated |
| IHC | 41.1 | 825 000 | 20 100 | 129 000 |

## Delayed colonoscopic surveillance for carriers of *MSH6* and *PMS2* mutations

It has been suggested, since *MSH6* and *PMS2* mutation carriers have a later age of onset and lower lifetime risk of colorectal cancer that it may be appropriate to start colonoscopic surveillance at age 45 instead of 25, thus saving costs and avoiding potential harms of colonoscopies.

As shown in Table 4 this does reduce incremental costs compared to the base case analysis, but incremental QALYs are also lost. Overall the cost-effectiveness of each testing strategy is slightly worsened. IHC with methylation remains the cost-effective strategy.

Table 4: Delayed surveillance for carriers of *MSH6* and *PMS2* mutations - cost-effectiveness results

| Strategy | Incremental QALYs vs. no testing | Incremental costs vs. no testing (£) | ICER vs. no testing (£/QALY) | Fully incremental ICER (£/QALY) |
| --- | --- | --- | --- | --- |
| MSI with methylation | 33.6 | 536 000 | 16 000 | Dominated |
| Direct mutation testing | 34.1 | 759 000 | 22 200 | Dominated |
| IHC with methylation | 36.9 | 527 000 | 14 300 | 14 300 |
| MSI | 37.3 | 761 000 | 20 400 | Extendedly dominated |
| IHC | 39.1 | 815 000 | 20 800 | 130 000 |

## Colonoscopy effectiveness estimates from Arrigoni et al. 2005

There is considerable uncertainty about the effectiveness of colonoscopic surveillance for reducing the incidence of CRC. While the base case estimate, from Jarvinen et al. 2000 [[11](#_ENREF_11)], is by no means the most optimistic estimate [[12](#_ENREF_12)], there is a study by Arrigoni et al. 2005 [[1](#_ENREF_1)] which estimates lower effectiveness.

When the effectiveness of surveillance colonoscopy is lowered, this unsurprisingly has a detrimental impact on the cost-effectiveness of testing strategies. In this case (see Table 5), while IHC with methylation testing remains cost-effective compared to the other testing strategies, it is no longer cost-effective compared to no testing at all. We further explored this finding by setting a number of other parameters to their “best case” values, including assuming 100% sensitivity and specificity of IHC and MSI, as well as 100% acceptance of counselling and mutation testing. In this case IHC with methylation was still not cost-effective compared to no testing, although the ICER was reduced to £27 900 per QALY.

This reinforces that the effectiveness of surveillance colonoscopy is critical for testing to be cost-effective, and that optimistic assumptions about other parameters are insufficient to support the case for testing if surveillance colonoscopy is indeed ineffective.

Table 5: Colonoscopy effectiveness estimates from Arrigoni et al. 2005 - cost-effectiveness results

| Strategy | Incremental QALYs vs. no testing | Incremental costs vs. no testing (£) | ICER vs. no testing (£/QALY) | Fully incremental ICER (£/QALY) |
| --- | --- | --- | --- | --- |
| Direct mutation testing | 10.0 | 805 000 | 80 200 | Dominated |
| MSI with methylation | 10.0 | 581 000 | 57 800 | Dominated |
| IHC with methylation | 11.0 | 577 000 | 52 500 | 52 500 |
| MSI | 11.5 | 810 000 | 70 400 | Extendedly dominated |
| IHC | 12.1 | 867 000 | 71 700 | 265 000 |

## MLH1 methylation testing performance estimated from Hampel et al. 2006

Only Hampel et al. 2006 [[2](#_ENREF_2)] conducted *MLH1* methylation testing in all patients with MSI-H tumours. As shown in Table 6, estimates from this study predict better performance in Lynch syndrome tumours caused by *MLH1* mutations, and in sporadic tumours, but substantially worse performance in LS tumours caused by other mutations.

Table 6: *MLH1* methylation testing probabilities following MSI-H

| Group | Probability of positive *MLH1* methylation test | |
| --- | --- | --- |
|  | Base case | Hampel et al. 2006 |
| LS: MLH1 mutation | 5% | 0% |
| LS: Other mutation | 7% | 22% |
| Sporadic | 67% | 79% |

The results of this scenario analysis are given in Table 7. In this scenario, MSI with methylation testing becomes the least effective option. It is still cost-effective compared to no testing, but is dominated by IHC with methylation.

Table 7: *MLH1* methylation testing probabilities from Hampel et al. 2006 - cost-effectiveness results

| Strategy | Incremental QALYs vs. no testing | Incremental costs vs. no testing (£) | ICER vs. no testing (£/QALY) | Fully incremental ICER (£/QALY) |
| --- | --- | --- | --- | --- |
| MSI with methylation | 34.5 | 545 000 | 15 800 | Dominated |
| Direct mutation testing | 35.1 | 769 000 | 21 900 | Dominated |
| IHC with methylation | 37.9 | 538 000 | 14 200 | 14 200 |
| MSI | 38.3 | 771 000 | 20 100 | Extendedly Dominated |
| IHC | 40.2 | 826 000 | 20 600 | 129 000 |

## Acceptance of genetic counselling estimated from Batte et al. 2014

In the base case analysis, it is assumed that 55% of women with tumour-based test results suggestive of Lynch syndrome will accept genetic counselling, based on a study by Heald et al. 2013 [[13](#_ENREF_13)]. Another study, by Batte et al. 2014 [[3](#_ENREF_3)], produces a higher estimate of 72%.

As expected, when the acceptance rate of genetic counselling is increased, the cost-effectiveness of testing is improved (Table 8).

Table 8: Acceptance of genetic counselling estimated from Batte et al. 2014 - cost-effectiveness results

| Strategy | Incremental QALYs vs. no testing | Incremental costs vs. no testing (£) | ICER vs. no testing (£/QALY) | Fully incremental ICER (£/QALY) |
| --- | --- | --- | --- | --- |
| Direct mutation testing | 35.1 | 769 000 | 21 900 | Dominated |
| MSI with methylation | 44.9 | 637 000 | 14 200 | Dominated |
| IHC with methylation | 49.4 | 627 000 | 12 700 | 12 700 |
| MSI | 49.9 | 941 000 | 18 900 | Extendedly dominated |
| IHC | 52.3 | 1 010 000 | 19 300 | 132 000 |

## Costs of CRC based on Murphy and Gray 2015

In this scenario, an alternative source for costs of CRC was used, which does not stratify according to age at diagnosis [[4](#_ENREF_4)]. Furthermore, these costs were substantially higher than those used in the base case. As expected, this improved the cost-effectiveness of testing strategies (Table 9), since preventing CRC saves more money in this scenario.

Table 9: Costs of CRC based on Murphy and Gray 2015 - cost-effectiveness results

| Strategy | Incremental QALYs vs. no testing | Incremental costs vs. no testing (£) | ICER vs. no testing (£/QALY) | Fully incremental ICER (£/QALY) |
| --- | --- | --- | --- | --- |
| MSI with methylation | 34.5 | 458 000 | 13 300 | Dominated |
| Direct mutation testing | 35.1 | 681 000 | 19 400 | Dominated |
| IHC with methylation | 37.9 | 442 000 | 11 600 | 11 600 |
| MSI | 38.3 | 673 000 | 17 600 | Extendedly dominated |
| IHC | 40.2 | 723 000 | 18 000 | 126 000 |

## Health state utility values relating to genetic counselling and testing based on Kuppermann et al. 2013

In this scenario analysis, utility multipliers were used based on the study by Kuppermann et al. 2013 [[5](#_ENREF_5)] as shown in Table 10.

Table 10: Health state utility value multipliers in scenario analysis

| Health state | Utility multiplier | Derivation |
| --- | --- | --- |
| Declining testing | 0.959 | Utility for declining testing = 0.745  Utility for negative test result = 0.777  Assume utility for negative test result = utility for someone not recommended genetic counselling |
| Declining counselling | 0.959 | Assumed equal to utility for declining testing |
| Diagnosed with LS pathogenic variant | 0.918 | Assumed twice as bad as declining testing |
| Diagnosed putative LS | 0.918 | Assumed equal to utility with LS diagnosis |

In this scenario analysis, IHC with *MLH1* methylation remains the only cost-effective strategy in a fully incremental analysis with a cost-effectiveness threshold of £20 000 per QALY, although it’s cost-effectiveness is slightly worsened, from a base case ICER of £14 200 per QALY to £15 100 per QALY.

Table 11: Health state utility values for genetic counselling and testing based on Kuppermann et al. 2013 - cost-effectiveness results

| Strategy | Incremental QALYs vs. no testing | Incremental costs vs. no testing (£) | ICER vs. no testing (£/QALY) | Fully incremental ICER (£/QALY) |
| --- | --- | --- | --- | --- |
| Direct mutation testing | 27.6 | 769 000 | 27 800 | Dominated |
| MSI with methylation | 32.0 | 545 000 | 17 100 | Dominated |
| MSI | 33.5 | 771 000 | 23 000 | Dominated |
| IHC | 35.0 | 826 000 | 26 700 | Dominated |
| IHC with methylation | 35.5 | 538 000 | 15 100 | 15 100 |

## Health state utility values for CRC based on Ness et al. 1999

Of the few studies which have investigated the impact of CRC stage on preference-based health utility values, most identify little difference between localised (Stage I/II), regional (Stage III) and metastatic (Stage IV) CRC [[14](#_ENREF_14)]. One study, by Ness et al. 1999 [[6](#_ENREF_6)], stands apart and identifies a very significant gradient for utility value according to disease stage. This study uses vignettes which include CRC-specific sequelae, such as faecal urgency and incontinence, which are not captured in generic preference-based utility measures such as EQ-5D.

As expected, a more significant gradient for utility value according to disease stage leads to testing for Lynch syndrome being more cost-effective (Table 12), since colonoscopic surveillance is effective at identifying CRC in earlier stages.

Table 12: Health state utility values for CRC based on Ness et al. 1999 - cost-effectiveness results

| Strategy | Incremental QALYs vs. no testing | Incremental costs vs. no testing (£) | ICER vs. no testing (£/QALY) | Fully incremental ICER (£/QALY) |
| --- | --- | --- | --- | --- |
| MSI with methylation | 35.8 | 535 000 | 15 200 | Dominated |
| Direct mutation testing | 36.4 | 769 000 | 21 100 | Dominated |
| IHC with methylation | 39.4 | 538 000 | 13 700 | 13 700 |
| MSI | 39.8 | 771 000 | 19 400 | Extendedly dominated |
| IHC | 41.7 | 826 000 | 19 800 | 125 000 |

## Risk prediction tools incorporated

As a scenario analysis we incorporated the risk prediction tools PREMM_1,2,6_, MMRpro and MMRpredict. We assumed they would have diagnostic performance as demonstrated in the population-based cohort in Mercado et al. 2012 [[7](#_ENREF_7)], specifically that their sensitivities and specificities would be as shown in Table 13. We assumed that MMRpro with tumour-based data would incorporate IHC information. We assumed a uniform cost of £116 to conduct risk prediction using any one of the prediction tools, based on the cost of a non-consultant-led outpatient appointment in gynaecologic oncology in the NHS [[15](#_ENREF_15)].

Table 13: Diagnostic performance of risk prediction tools

| Prediction tool | Sensitivity [%] | Specificity [%] |
| --- | --- | --- |
| PREMM_1,2,6_ | 64.3 | 85.2 |
| MMRpredict | 71.4 | 63.8 |
| MMRpro | 57.1 | 84.9 |
| MMRpro + IHC | 64.3 | 89.0 |

In this scenario analysis the risk prediction tools all produced fewer QALYs than the existing testing strategies, and all were dominated or extendedly dominated, as shown in Table 14. IHC with methylation testing remained the cost-effective strategy.

Table 14: Risk prediction tools incorporated – cost-effectiveness results

| Strategy | Incremental QALYs vs. no testing | Incremental costs vs. no testing (£) | ICER vs. no testing (£/QALY) | Fully incremental ICER (£/QALY) |
| --- | --- | --- | --- | --- |
| MMRpro | 22.9 | 389 000 | 17 000 | Extendedly dominated |
| PREMM_1,2,6_ | 25.7 | 400 000 | 15 600 | Extendedly dominated |
| MMRpro + IHC | 26.8 | 586 000 | 21 800 | Dominated |
| MMRpredict | 29.2 | 628 000 | 21 500 | Dominated |
| MSI with methylation | 34.5 | 545 000 | 15 800 | Dominated |
| Direct mutation testing | 35.1 | 769 000 | 21 900 | Dominated |
| IHC with methylation | 37.9 | 538 000 | 14 200 | 14 200 |
| MSI | 38.3 | 771 000 | 20 100 | Extendedly dominated |
| IHC | 40.2 | 826 000 | 20 600 | 129 000 |

## Cost of diagnostic mutation testing £525 following IHC

As a scenario analysis we explored the impact of diagnostic mutation testing being cheaper following IHC as not all genes need to be tested in the vast majority of cases. We assumed (based on information from the UK Genetic Testing Network) that diagnostic mutation testing would cost £525 as this is the median cost for two-gene panels which include *MLH1* and *PMS2* or *MSH2* and *MSH6*.

In this scenario analysis the costs of IHC with methylation testing were marginally lower (£10 per patient) and the costs of IHC without methylation testing were £32 lower. All other results were unchanged, as shown in Table 15. IHC with methylation testing remained the cost-effective strategy.

Table 15: Risk prediction tools incorporated – cost-effectiveness results

| Strategy | Incremental QALYs vs. no testing | Incremental costs vs. no testing (£) | ICER vs. no testing (£/QALY) | Fully incremental ICER (£/QALY) |
| --- | --- | --- | --- | --- |
| MSI with methylation | 34.5 | 545 000 | 15 800 | Dominated |
| Direct mutation testing | 35.1 | 769 000 | 21 900 | Dominated |
| IHC with methylation | 37.9 | 527 000 | 13 900 | 13 900 |
| MSI | 38.3 | 771 000 | 20 100 | Extendedly dominated |
| IHC | 40.2 | 794 000 | 19 800 | 120 000 |

# References

1. Arrigoni A, Sprujevnik T, Alvisi V, Rossi A, Ricci G, Pennazio M, et al. Clinical identification and long-term surveillance of 22 hereditary non-polyposis colon cancer Italian families. Eur J Gastroenterol Hepatol. 2005;17(2):213-9. Epub 2005/01/28. PubMed PMID: 15674100.

2. Hampel H, Frankel W, Panescu J, Lockman J, Sotamaa K, Fix D, et al. Screening for Lynch syndrome (hereditary nonpolyposis colorectal cancer) among endometrial cancer patients. Cancer Res. 2006;66(15):7810-7. Epub 2006/08/04. doi: 10.1158/0008-5472.CAN-06-1114. PubMed PMID: 16885385.

3. Batte BAL, Bruegl AS, Daniels MS, Ring KL, Dempsey KM, Djordjevic B, et al. Consequences of universal MSI/IHC in screening endometrial cancer patients for Lynch syndrome. Gynecol Oncol. 2014;134(2):319-25. doi: 10.1016/j.ygyno.2014.06.009.

4. Murphy J, Gray A. The cost-effectiveness of immunochemical faecal occult blood testing vs. guaiac faecal occult blood testing for colorectal cancer screening in the NHS Bowel Cancer Screening Programme. University of Oxford: Health Economics Research Centre, 2015.

5. Kuppermann M, Wang G, Wong S, Blanco A, Conrad P, Nakagawa S, et al. Preferences for outcomes associated with decisions to undergo or forgo genetic testing for Lynch syndrome. Cancer. 2013;119(1):215-25. Epub 2012/07/13. doi: 10.1002/cncr.27634. PubMed PMID: 22786716; PubMed Central PMCID: PMC4356667.

6. Ness RM, Holmes AM, Klein R, Dittus R. Utility valuations for outcome states of colorectal cancer. Am J Gastroenterol. 1999;94(6):1650-7. Epub 1999/06/11. doi: 10.1111/j.1572-0241.1999.01157.x. PubMed PMID: 10364039.

7. Mercado RC, Hampel H, Kastrinos F, Steyerberg E, Balmana J, Stoffel E, et al. Performance of PREMM(1,2,6), MMRpredict, and MMRpro in detecting Lynch syndrome among endometrial cancer cases. Genet Med. 2012;14(7):670-80. Epub 2012/03/10. doi: 10.1038/gim.2012.18. PubMed PMID: 22402756; PubMed Central PMCID: PMC3396560.

8. Moller P, Seppala T, Bernstein I, Holinski-Feder E, Sala P, Evans DG, et al. Incidence of and survival after subsequent cancers in carriers of pathogenic MMR variants with previous cancer: a report from the prospective Lynch syndrome database. Gut. 2017;66(9):1657-64. Epub 2016/06/05. doi: 10.1136/gutjnl-2016-311403. PubMed PMID: 27261338; PubMed Central PMCID: PMC5561364.

9. Moller P, Seppala T, Bernstein I, Holinski-Feder E, Sala P, Evans DG, et al. Cancer incidence and survival in Lynch syndrome patients receiving colonoscopic and gynaecological surveillance: first report from the prospective Lynch syndrome database. Gut. 2017;66(3):464-72. Epub 2015/12/15. doi: 10.1136/gutjnl-2015-309675. PubMed PMID: 26657901; PubMed Central PMCID: PMC5534760.

10. Moller P, Seppala TT, Bernstein I, Holinski-Feder E, Sala P, Evans DG, et al. Cancer risk and survival in path_MMR carriers by gene and gender up to 75 years of age: a report from the Prospective Lynch Syndrome Database. Gut. 2017. Epub 2017/07/30. doi: 10.1136/gutjnl-2017-314057. PubMed PMID: 28754778.

11. Jarvinen HJ, Aarnio M, Mustonen H, Aktan-Collan K, Aaltonen LA, Peltomaki P, et al. Controlled 15-year trial on screening for colorectal cancer in families with hereditary nonpolyposis colorectal cancer. Gastroenterology. 2000;118(5):829-34. Epub 2000/04/28. PubMed PMID: 10784581.

12. Ladabaum U, Ford JM, Martel M, Barkun AN. American Gastroenterological Association Technical Review on the Diagnosis and Management of Lynch Syndrome. Gastroenterology. 2015;149(3):783-813 e20. Epub 2015/08/01. doi: 10.1053/j.gastro.2015.07.037. PubMed PMID: 26226576.

13. Heald B, Plesec T, Liu X, Pai R, Patil D, Moline J, et al. Implementation of universal microsatellite instability and immunohistochemistry screening for diagnosing lynch syndrome in a large academic medical center. J Clin Oncol. 2013;31(10):1336-40. Epub 2013/02/13. doi: 10.1200/JCO.2012.45.1674. PubMed PMID: 23401454; PubMed Central PMCID: PMC4878100.

14. Jeong K, Cairns J. Systematic review of health state utility values for economic evaluation of colorectal cancer. Health Econ Rev. 2016;6(1):36. Epub 2016/08/20. doi: 10.1186/s13561-016-0115-5. PubMed PMID: 27541298; PubMed Central PMCID: PMC4991979.

15. Department of Health. NHS reference costs 2017 to 2018. 2018 [cited 2019 April 23]. Available from: <https://improvement.nhs.uk/resources/reference-costs/>.
