# Supplementary material for: Cost-effectiveness analysis of reflex testing for Lynch syndrome in women with endometrial cancer in the UK setting
Source: PLoS One. 2019 Aug 30;14(8):e0221419. doi: 10.1371/journal.pone.0221419 (PMC6716649; doi:10.1371/journal.pone.0221419)
Supplement: S7 Appendix — (DOCX) [file pone.0221419.s007.docx]

# S7 Appendix. Estimation of life expectancy according to age and other risk factors

Life expectancy is an important clinical outcome to predict, although for economic evaluations it is not preferred because it does not include discounting (time preference) or adjustment for quality of life as QALYs do.

The model was used to estimate life expectancy for probands and relatives according to the age at which they are diagnosed and compare this to the counterfactual outcome if they did not receive a diagnosis. There are four possible diagnostic outcomes:

- False negative: The individual truly has Lynch syndrome but does not receive a diagnosis of Lynch syndrome;
- Putative Lynch syndrome: The individual may or may not have Lynch syndrome and no causative mutation has been identified but they are recommended to receive surveillance in accordance with a diagnosis of Lynch syndrome;
- True positive: The individual truly has Lynch syndrome and the causative mutation has been identified;
- True negative: The individual does not have Lynch syndrome and they are not diagnosed with Lynch syndrome or recommended to receive intensive surveillance.

The results are shown in Figure 1 and Figure 2 and indicate the following findings:

- Older individuals have lower life expectancy (as is to be expected);
- Female relatives have higher life expectancy than male relatives;
- Life expectancy is lowest for probands who do not have Lynch syndrome (because survival of sporadic endometrial cancer is worse than survival of Lynch syndrome-associated endometrial cancer);
- Life expectancy is highest for relatives who do not have Lynch syndrome (because they do not have an increased risk of colorectal cancer);
- True positive diagnosis is the best possible outcome for those with Lynch syndrome, while false negative diagnosis is the worst;
- A putative Lynch syndrome diagnosis does not materially affect the life expectancy for people who do not actually have Lynch syndrome;
- The biggest potential life expectancy gain is for individuals with *MLH1* or *MSH2* Lynch syndrome mutations.


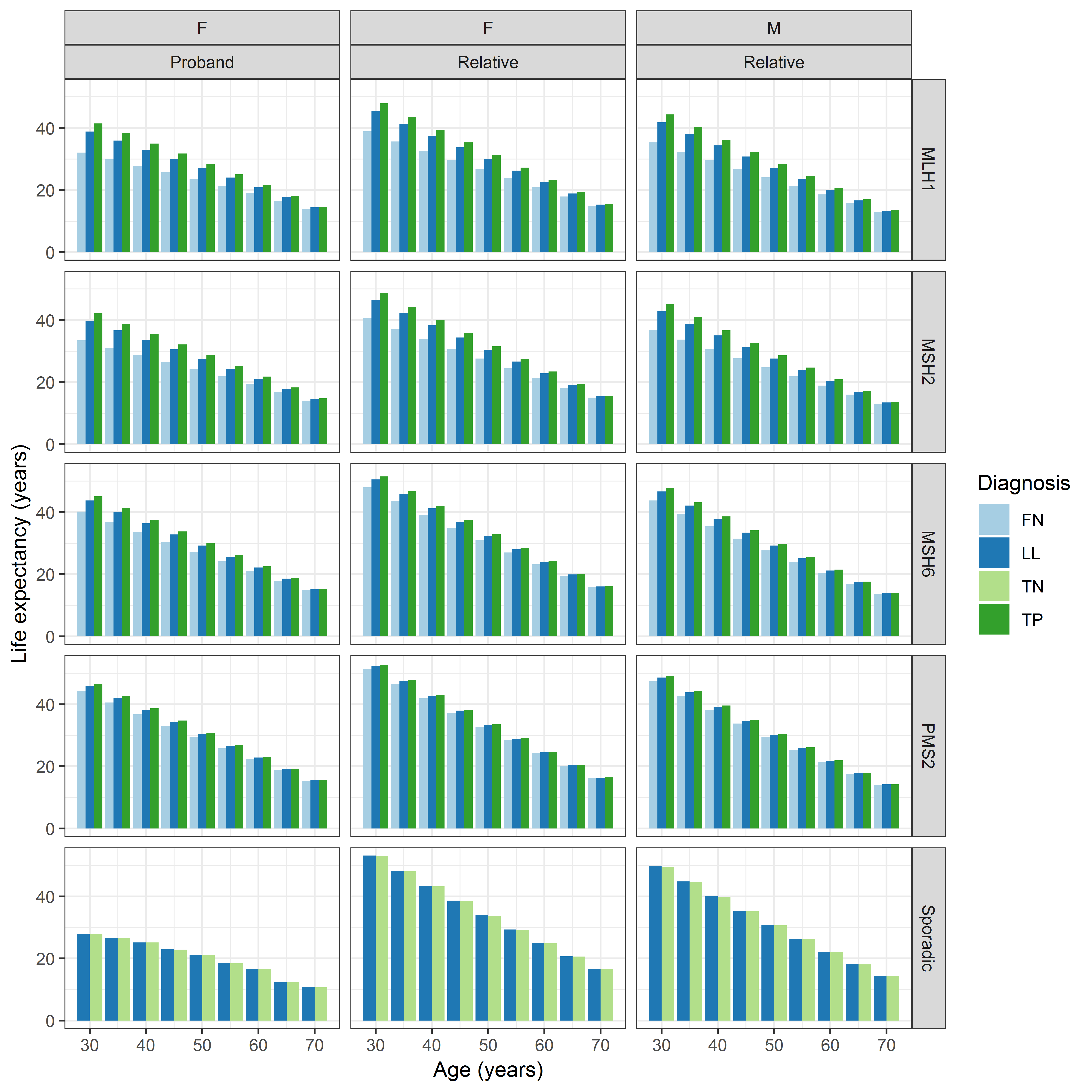


Figure 1: Life expectancy predicted by the model

Key: F, female; FN, false negative; LL, likely (putative) Lynch syndrome; M, male; TN, true negative; TP, true positive


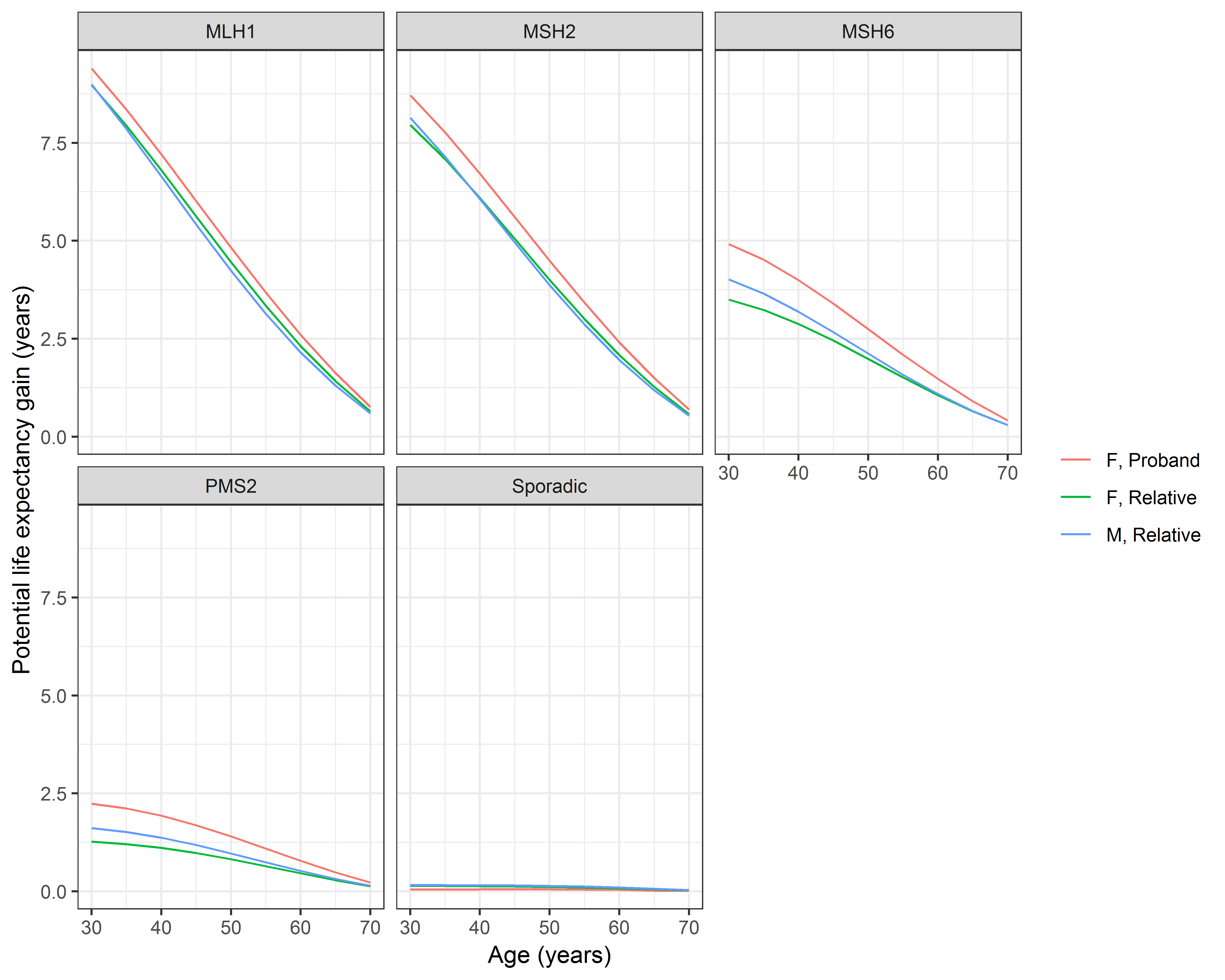


Figure 2: Potential life expectancy gain predicted by the model

Key: F, female; M, male
